# Association of Physical Activity With Risk of Major Cardiovascular Diseases in Chinese Men and Women

**DOI:** 10.1001/jamacardio.2017.4069

**Published:** 2017-11-08

**Authors:** Derrick A. Bennett, Huaidong Du, Robert Clarke, Yu Guo, Ling Yang, Zheng Bian, Yiping Chen, Iona Millwood, Canqing Yu, Pan He, Xiangyang Zheng, Rory Collins, Junshi Chen, Richard Peto, Liming Li, Zhengming Chen

**Affiliations:** 1 Clinical Trial Service Unit and Epidemiological Studies Unit, Nuffield Department of Population Health, University of Oxford, Oxford, England; 2 Medical Research Council Population Health Research Unit, Nuffield Department of Population Health, University of Oxford, Oxford, England; 3 Chinese Academy of Medical Sciences, Beijing, China; 4 Department of Epidemiology and Biostatistics, School of Public Health, Peking University Health Science Center, Beijing, China; 5 NCDs Prevention and Control Department, Huixian Centers for Disease Control and Prevention, Xianxiang, China; 6 NCDs Prevention and Control Department, Meilan Centers for Disease Control and Prevention, Haikou, China; 7 China National Center For Food Safety Risk Assessment, Beijing, China

## Abstract

**Importance:**

In China, the patterns and levels of physical activity differed from those in high-income countries. Substantial uncertainty remains about the relevance, both qualitatively and quantitatively, of domain-specific physical activity for cardiovascular disease (CVD) subtypes in Chinese adults.

**Objective:**

To assess the shape and strength of the associations of total, occupational, and nonoccupational physical activity with CVD subtypes in Chinese men and women.

**Design, Setting, and Participants:**

This population-based prospective cohort study in 10 (5 urban, 5 rural) areas across China included 487 334 adults who were aged 30 to 79 (mean 51) years with no prior CVD history when enrolled from June 2004 to July 2008.

**Exposures:**

Self-reported total, occupational, and nonoccupational physical activity, quantified as metabolic equivalent of task hours per day (MET-h/d) based on the type, frequency, and duration of specific activities.

**Main Outcomes and Measures:**

Major vascular events (n = 36 184) and their components, including major coronary events (n = 5082), ischemic stroke (n = 25 647), intracerebral hemorrhage (n = 5252), and CVD death (n = 8437). Cox regression yielded adjusted hazard ratios for each disease that was associated with physical activity.

**Results:**

Of the 487 334 study participants, 287 527 (59%) were women and the mean (SD) age was 51 (10.5) years. The overall mean (SD) total physical activity was 21.5 (12.8) MET-h/d, mainly from occupational activity, especially among men (75% vs 50% in women). Total physical activity was inversely associated with the risk of major vascular events, with the adjusted hazard ratio that compared the top vs bottom quintiles of physical activity being 0.77 (95% CI, 0.74-0.80). Throughout the range of total physical activity studied, the association with CVD with each 4 MET-h/d higher usual total physical activity (approximately 1 hour of brisk walking per day) associated with a 6% (95% CI, 5%-7%) lower risk of major vascular events, and a 9%, 5%, 6%, and 12% lower risk of major coronary events, ischemic stroke, intracerebral hemorrhage, and CVD death, respectively. The strength of the associations was similar and independent of each other for occupational and nonoccupational physical activity. However, for occupational physical activity, the associations with CVD subtypes were greatly attenuated above 20 MET-h/d, especially for intracerebral hemorrhage. The associations of total physical activity with major vascular events were similar among men and women and across different levels of sedentary leisure time but were much weaker among individuals with high blood pressure.

**Conclusions and Relevance:**

Among Chinese adults, higher occupational or nonoccupational physical activity was associated with significantly lower risks of major CVD.

## Key Points

**Question:**

What are the shape and strength of the associations between physical activity and risks of major cardiovascular diseases (CVDs)?

**Findings:**

In a 7.5-year study of 487 334 adults without prior CVD from 10 areas in China, total, occupational, and nonoccupational physical activity showed an inverse dose-response association with risk of CVD, with each 4 metabolic equivalent of task hours per day higher activity (approximately 1 hour per day cycling or brisk walking) associated with a 5% to 12% lower risk of developing various types of CVD.

**Meaning:**

Among Chinese adults, higher physical activity may be associated with lower CVD risk, reinforcing current guidelines that promote any physical activity for CVD prevention.

## Teaser

This prospective study explores the association between higher levels of physical activity and all major subtypes of cardiovascular disease in Chinese men and women.

## Introduction

Low levels of physical activity are an important modifiable risk factor for cardiovascular disease (CVD).^1^ In recent decades, levels of physical activity have declined worldwide, including in China, mainly because of rapid urbanization, reduced physical activity in the work place, changes in modes of transportation, and other aspects of lifestyle.^2^ The Global Burden of Diseases report estimated that low levels of physical activity accounted for 2.1 million premature deaths and 4.5 million disability-adjusted life-years worldwide in 2013.^3^ These estimates were mainly based on studies in high-income countries where patterns and levels of physical activity differed appreciably from those in low-income and middle-income countries, including China.^4^ Within China there are also large differences in physical activity levels between urban and rural areas.^5^ Previous studies of physical activity levels in China have been constrained by a small number of events^6^ that considered only fatal outcomes,^7^ covered a single geographic area,^8^ reported associations with CVD risk factors only,^9^ or had a combination of these constraints.^10^

Total physical activity is composed of occupational, commuting, household, and leisure time physical activity. Higher levels of both occupational and leisure time physical activity have been associated with lower risks of CVD in high-income countries.^11^ There is evidence that higher levels of physical activity may also offset the risks of CVD that are associated with high levels of sedentary leisure time.^12^ In China, there has been a substantial shift from labor-intensive lifestyles to more sedentary lifestyles in recent decades,^14^ but the relevance of occupational and nonoccupational (including leisure time) physical activity for risks of subtypes of CVD, both overall and among different population subgroups (eg, different ages or levels of blood pressure), is lacking.^15^ Moreover, the disease patterns in China differ importantly from those in the high-income countries (eg, there are higher rates of stroke than ischemic heart disease [IHD] in China) and also vary significantly between regions.^4^ Reliable assessment of the association of physical activity levels with different subtypes of CVD (particularly stroke) in China is needed to inform disease prevention programs. Based on data from a large prospective study of Chinese adults, the present study aimed to (1) quantify the associations of total, occupational, and nonoccupational physical activity with the risks of several major subtypes of CVD; (2) assess whether these associations differed by age, sex, and different levels of baseline risk factors (eg, blood pressure); and (3) examine the joint effects of total physical activity levels with sedentary leisure time and of occupational with nonoccupational physical activity.

## Methods

### Study Population

Details of the study design and methods of the China Kadoorie Biobank (CKB) have been previously reported.^18^ Briefly, participants were recruited from 10 (5 urban and 5 rural) diverse areas in China, chosen from China’s nationally representative Disease Surveillance Points (DSP) system^21^ to maximize geographic and socioeconomic diversity (eFigure 1 in the Supplement). All 1 801 200 registered residents who were thought to be 35 to 74 years in the study areas were identified through local residential records and invited to attend study clinics between June 2004 and July 2008. Overall, 512 891 (the response rate was around 30%) participated, including 12 665 who were just outside this age range (making the actual baseline age range from 30-79 years). Individuals with prior physician-diagnosed IHD (n = 15 472), those who had experienced a stroke/transient ischemic attack (n = 8884), and those that reported implausibly small, large, or conflicting levels of physical activity (n = 10 201) were excluded, leaving 487 334 participants for this analysis.

Trained health workers administered a laptop-based questionnaire on demographic, socioeconomic, and lifestyle factors (eg, smoking, alcohol drinking, diet, physical activity levels, and medical history) and measured height, weight, waist and hip circumference, blood pressure, and lung function. Blood samples were collected for the onsite testing of random plasma glucose levels (SureStep Plus System, Johnson and Johnson) and then separated into plasma and buffy coat fractions for long-term storage. Periodic resurveys were conducted on approximately 25 000 (5%) of the randomly selected surviving participants. Before the study began, ethical approval was obtained from local, national, and international ethics committees. All participants provided written informed consent.

### Physical Activity Measurements

The details of the methods that were used to assess physical activity have been previously reported^16^ and were comparable with the methods that were used in previous studies in both high-income^23^ and Chinese^24^ populations. The CKB baseline physical activity questionnaire covered relevant questions on the intensity, frequency, and time spent on occupational tasks, commuting, household tasks, and leisure time activities (see detailed questionnaire at http://www.ckbiobank.org), but has not been compared directly with a reference method, such as an accelerometer. Metabolic equivalents of task (METs) of different types of activities were adopted from the 2011 compendium of physical activities^25^ (eTable 1 in the Supplement). Occupational physical activity included all physical activity that was performed during paid employment. Nonoccupational physical activity included all physical activity that was performed during travel to and from work, household activity, and leisure-time activity (excluding sedentary leisure time activities, such as watching television or reading).^16^ The MET of each activity was subsequently multiplied by the frequency and duration of physical activity to calculate total physical activity in MET hours per day (MET-h/d).

### Follow-up for Morbidity and Mortality Rates

All 10 study regions are part of DSP system, which provides mortality rate statistics for the entire country.^21^ The vital status of all participants was monitored periodically through DSP death registries as well as health insurance systems and was supplemented by annual active confirmation of survival through local street committees or village administrators. The underlying causes of death were coded by trained DSP staff members who used *International Statistical Classification of Diseases and Related Health Problems, Tenth Revision (ICD-10)*^26^ and were blinded to the baseline information. Data on nonfatal disease outcomes were obtained by linkage, using each participant’s personal identification number, with established local chronic disease registries (eg, IHD and stroke) and with the National Health Insurance claim system, which provided almost universal (approximately 99%) coverage of all hospitalized events in all study regions. By January 1, 2015, 25 488 participants (5%) had died and 2411 (0.4%) were lost to follow-up. 

The primary outcomes examined in the present study were first major coronary events (MCEs) (fatal IHD [*ICD-10*: I20-I25] or nonfatal myocardial infarction [I21-I23]), ischemic stroke (IS) (I63), intracerebral hemorrhage (ICH) (I61), cardiovascular death (I00-I99), and major vascular events (MVEs) (any of the previously mentioned events plus other types of nonfatal stroke [I60 and I64]).

### Statistical Analyses

Selected characteristics of the study participants were compared by quintiles of total physical activity, after adjusting for age (5-year groups), sex, and region using linear or logistic regression when appropriate. Cox regression analysis was used to obtain hazard ratios (HRs) for CVD events that were associated with quintiles of total physical activity. Analyses were stratified by age at risk (5-year intervals), sex, and region (10 groups) and adjusted for household income, education, alcohol consumption (all used 6 groups), smoking (4 groups), fresh fruit intake (5 groups), sedentary leisure time, and self-reported general health status (4 groups). Analyses of occupational and nonoccupational physical activity levels also included additional mutual adjustment for each other. The HRs and 95% CIs for quintiles of physical activity were computed using floating absolute risks (such that the HR in each group, including the one for the reference group, was associated with a group-specific 95% CI that reflected the amount of data in that category).^27^ The proportional hazards assumptions for the Cox model were assessed using standard methods and were satisfied.^28^

The combined effects of measurement error and within-person variability mean that using a single measurement of physical activity at baseline can lead to a substantial underestimation (ie, “regression dilution bias”) of the importance of physical activity levels to disease risk.^29^ Therefore, risk estimates were corrected for regression dilution bias using physical activity–level data collected at resurvey among approximately 20 000 participants (conducted around 3 years after the baseline survey). The regression dilution ratios (RDRs) were calculated using the McMahon-Peto method, which uses the ratio of the ranges (top vs bottom quintile, defined by baseline physical activity) of the mean physical activity levels at resurvey to the range of such measurements at baseline.^30^ For comparison, we also computed RDRs using the correlation between measurements for participants with baseline and resurvey data.^31^ A departure from linearity was assessed using the likelihood ratio test and log HRs per 4 MET-h/d higher baseline physical activity levels were then multiplied by the reciprocal of the RDRs to obtain HRs (and associated 95% CI) for the usual physical activity level. Physical activity results were presented as the HRs per 4 MET-h/d higher usual physical activity (equivalent to 1 hour of walking per day) with CVD.

The joint effects of total physical activity levels and sedentary leisure time were assessed by creating 9 groups based on tertiles, of both total physical activity and sedentary leisure time, using Cox regression to estimate the HRs of MVE for each group. Likewise, the joint effects of tertiles of occupational and nonoccupational physical activity were also assessed by similar methods.

Sensitivity analyses excluded participants who reported other chronic diseases, poor self-rated general health at baseline, and CVD events that occurred during the first 3 years of follow-up. In addition, we examined the effect of sequential adjustment for many lifestyle factors, dietary factors, and physical measurements. Analyses used 2-sided *P* values without any correction for multiple testing, but allowances for multiple comparisons were made by using 99% CIs for subgroup analyses. Statistical significance was set at *P* = .05. All analyses were conducted using SAS, version 9.2 (SAS Institute) and R, version 3.0.1 (R Foundation).

## Results

### Baseline Characteristics of Participants by Physical Activity

Among the 487 334 participants, the mean (SD) age at baseline was 51 (10.5) years, 287 527 (59%) were women, and 204 680 (42%) resided in urban areas (Table). Individuals with higher total physical activity levels were more likely to be male, younger, and living in rural areas, and have lower levels of education than those with lower physical activity levels. Likewise, higher total physical activity was also associated with better health (eg, less diabetes or hypertension); lower blood pressure, heart rates, and body mass index (calculated as weight in kilograms divided by height in meters squared); and more favorable blood biochemistry profiles (eg, lower low-density lipoprotein cholesterol, C-reactive protein, cystatin C) (eTable 2 in the Supplement).

**Table.** Selected Characteristics for All Participants by Levels of Physical Activity^a^

Characteristic	Total Physical Activity (MET-h/d)					All
	≤9.1	9.2-14.7	14.8-22.4	22.5-3.7	≥33.8	
No. of participants	97 454	97 520	97 475	97 424	97 461	487 334
Physical activity–related factors, mean (SD)						
Total physical activity, MET-h/d	6.1 (4.4)	12.0 (4.3)	18.3 (4.3)	27.7 (4.3)	43.3 (4.4)	21.5 (12.8)
Occupational physical activity, MET-h/d	1.3 (6.0)	3.9 (5.8)	9.4 (5.8)	18.3 (5.8)	33.8 (5.9)	13.4 (11.5)
Nonoccupational physical activity, MET-h/d	4.8 (4.3)	8.1 (4.1)	8.9 (4.1)	9.4 (4.1)	9.5 (4.2)	8.2 (4.1)
Sedentary leisure time, h/d	3.5 (1.5)	3.3 (1.4)	2.9 (1.4)	2.7 (1.4)	2.6 (1.5)	3.0 (1.4)
Demographic factors						
Age, mean (SD), y	57.9 (9.8)	54.2 (9.8)	49.9 (9.7)	47.6 (9.8)	45.7 (10.0)	51.1 (10.5)
Female, No. (%)	53895 (59.2)	66837 (70.7)	61372 (62.6)	56083 (55.0)	50129 (48.1)	288014 (59.1)
Urban, No. (%)	53030 (54.5)	45995 (47.2)	44306 (45.4)	36020 (36.8)	31407 (32.0)	205655 (42.2)
Socioeconomic and lifestyle factors, No. (%)						
≥High school	19145 (18.7)	21456 (22.0)	27110 (27.3)	20307 (20.4)	13318 (15.4)	101365 (20.8)
Household income >20 000 ¥ (US $3006.30)/y	35290 (37.2)	40544 (45.0)	45852 (47.7)	39958 (43.0)	47062 (41.0)	208579 (42.8)
Male current smoker	57.2	60.3	61.3	64.7	66.3	62.0
Female current smoker	2.1	1.8	2.8	2.9	1.7	2.3
Male current drinker	30.2	35.6	35.8	33.7	34.2	33.9
Female current drinker	1.6	1.8	2.7	2.5	1.7	2.1
Regular fresh fruit consumption (>4 d/week)	28.7	30.8	30.2	24.5	24.4	27.7
Physical and blood measurements, mean (SD)						
SBP, mm Hg	131.6 (21.5)	131.8 (20.8)	130.8 (20.3)	130.8 (20.8)	130.8 (21.5)	131.1 (21.8)
DBP, mm Hg	78.5 (12.4)	78.7 (12.0)	78.2 (11.7)	77.9 (12.0)	77.6 (12.4)	78.2 (11.8)
Heart rate, bpm	80.0 (12.5)	79.4 (12.0)	78.7 (11.7)	78.5 (12.0)	77.9 (12.5)	78.9 (11.8)
BMI	23.8 (3.5)	23.9 (3.3)	23.5 (3.3)	23.4 (3.3)	23.4 (3.5)	23.6 (3.4)
FEV1/FVC, %	84.3 (8.0)	84.7 (7.8)	84.7 (7.6)	84.6 (7.8)	84.4 (8.0)	84.5 (8.5)
Random plasma glucose level, mg/dL^b^	111.1 (43.1)	109.3 (41.6)	107.8 (40.6)	107.5 (41.7)	107.4 (43.1)	108.7 (41.1)
Self-reported conditions at baseline, No. (%)						
Diabetes	35290 (3.9)	40544 (3.4)	2250 (2.7)	1379 (2.0)	1032 (1.6)	13158 (2.7)
Hypertension	14416 (11.2)	12505 (11.7)	8611 (9.9)	6751 (8.9)	5972 (7.9)	48246 (9.9)
Poor health	12374 (13.5)	9604 (9.7)	8500 (8.3)	8410 (8.1)	6726 (7.5)	45809 (9.4)

Abbreviations: BMI, body mass index (calculated as weight in kilograms divided by height in meters squared); DBP, diastolic blood pressure; FEV, forced expiratory volume; FVC, forced vital capacity; MET-h/d, metabolic equivalents of task per hours per day; SBP, systolic blood pressure.

SI conversion factor: to convert glucose to millimoles per liter, muliply by 0.0555.

^a^ All values, means, and percentages are adjusted for age, sex, and region when appropriate.

^b^ Excluding 8151 participants with missing data.

Mean (SD) levels of total physical activity were higher among men than women (22.9 [12.8] vs 20.6 [12.8] MET-h/d, respectively), with men having higher occupational (17.4 [12.4] vs 10.6 [12.4] MET-h/d, respectively) but lower nonoccupational physical activity levels (5.5 [4.4] vs 10.0 [4.4] MET-h/d, respectively) than women (eTable 3 in the Supplement). Thus, occupational physical activity accounted for a higher proportion of total physical activity in men than in women (75% vs 50%, respectively) and in those living in rural areas rather than urban areas (65% vs 58%, respectively) (eTable 3 in the Supplement). By contrast, the time engaged in sedentary leisure time activity was similar among men and women (3.1 vs 2.9 hours per day, respectively, in both urban and rural areas) (eTable 3 in the Supplement).

### Associations of Total Physical Activity With Major Vascular Disease Events

**Figure 1.**
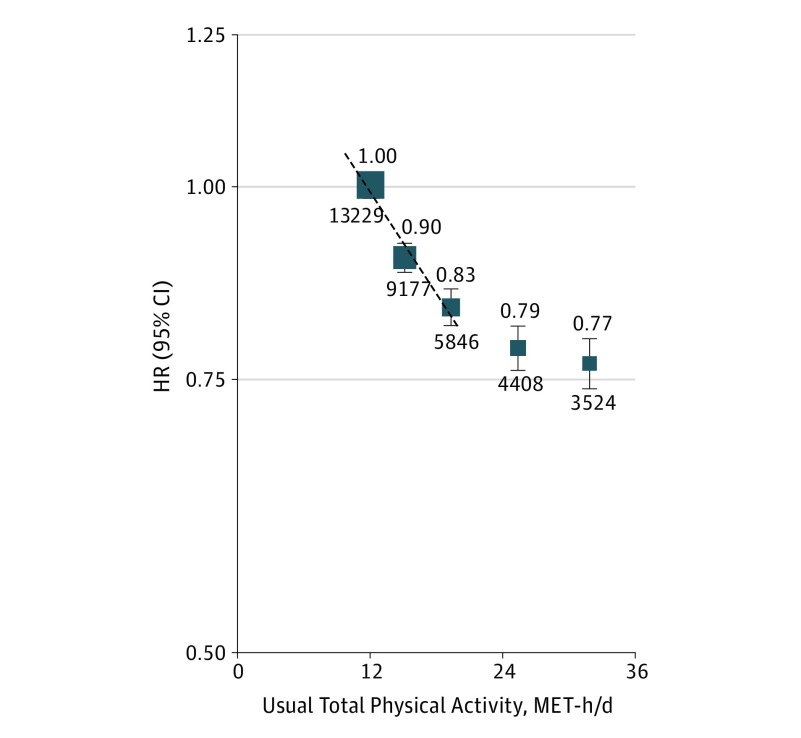
Adjusted Hazard Ratios (HRs) for Major Vascular Events by Total Physical Activity Values shown are the HR (95% CI) for major vascular events (n = 36 184) by quintiles of total physical activity after adjustment for age, sex, region, household income, education, alcohol consumption, smoking, fresh fruit intake, sedentary leisure time, and self-reported general health status. The size of the squares is proportional to the inverse variance of each effect size. The dashed line represents the slope from a weighted linear regression with weights based on the inverse variance of the log hazard ratios. The hazard ratio per 4 usual metabolic equivalent of task hours per day (MET-h/d) was 0.94 (95% CI, 0.93-0.95).

**Figure 2.**
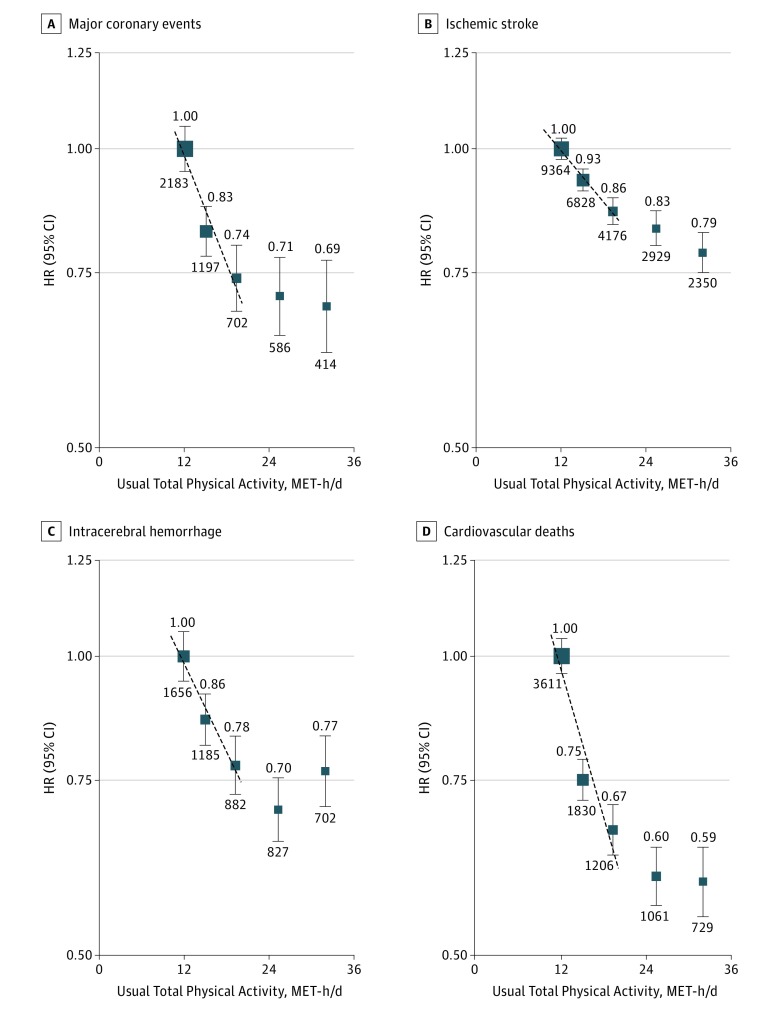
Adjusted Hazard Ratios (HRs) for Major Cardiovascular Diseases by Total Physical Activity Adjusted HRs for major coronary events (n = 5082; HR per 4 usual metabolic equivalent of task hours per day [MET-h/d], 0.91 [95% CI, 0.89-0.93]) (A), ischemic stroke (n = 25 647; HR per 4 usual MET-h/d, 0.95 [95% CI, 0.94-0.96]) (B), intracerebral hemorrhage (n = 5252; HR per 4 usual MET-h/d, 0.94 [95% CI, 0.92-0.96]) (C), and cardiovascular deaths (n = 8437; HR per 4 usual MET-h/d, 0.88 [95% CI, 0.86-0.90]) (D). Symbols and conventions as in Figure 1.

During 3.8 million person-years of follow-up, there were 36 184 MVEs, including 5082 MCE, 25 647 IS, 5252 ICH, and 8437 participants who died of CVD. Total physical activity levels were approximately log-linearly and were inversely associated with the risk of MVE in the range of about 10 to 20 MET-h/d (Figure 1). Individuals in the top quintile of total physical activity had a 23% lower risk of developing MVE (HR, 0.77; 95% CI, 0.74-0.80) compared with those in the bottom quintile. Likewise, total physical activity levels were linearly and inversely associated with each subtype of MVE (except for ICH, for which the association was attenuated at higher levels of total physical activity) (Figure 2C). For specific types of CVD, the adjusted HRs for the top vs bottom quintile of baseline total physical activity were 0.69 (95% CI, 0.62-0.77) for MCE, 0.79 (95% CI, 0.75-0.82) for IS, 0.77 (95% CI, 0.71-0.83) for ICH, and 0.59 (0.55-0.64) for CVD death (Figure 2; eTable 4 in the Supplement). The analyses of event rates by quintile of physical activity showed generally lower event rates by outcome for total and nonoccupational physical activity (eTable 5 in the Supplement).

The estimated RDRs were 0.53 for total (0.63 and 0.50 for occupational and nonoccupational physical activity, respectively) physical activity and the correlation results were broadly similar (eTable 6 in the Supplement). By applying these RDRs, each 4 usual MET-h/d higher levels of total physical activity was associated with 6%, 9%, 5%, 6%, and 12% lower risks of MVE, MCE, IS, ICH, and CVD death, respectively (Figure 1 and Figure 2). For each outcome, the shape and strength of these associations across the range of total physical activity were broadly similar for residents who were living in urban and rural areas and among men and women (eFigures 2-6 in the Supplement).

### Associations of Occupational and Nonoccupational Activity With Major Vascular Disease Events

**Figure 3.**
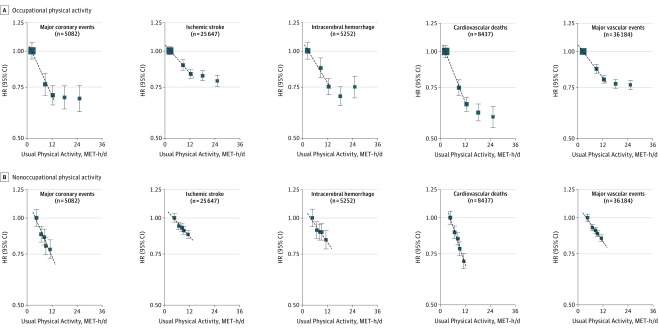
Adjusted Hazard Ratios (HRs) for Major Cardiovascular Diseases by Occupational and Nonoccupational Physical Activity Symbols and conventions as in Figure 1. Occupational and nonoccupational physical activity are mutually adjusted for each other.

Within the range of 2 to 12 MET-h/d, the shape and strength of the inverse associations with specific types of CVD were similar for occupational and nonoccupational physical activity (Figure 3; eTable 4 in the Supplement). For occupational physical activity, there was a flattening of the inverse association at approximately 20 MET-h/d for all outcomes, while for ICH the association was greatly attenuated above 20 MET-h/d (Figure 3A). For nonoccupational physical activity, there was an approximately log-linear inverse dose-response relationship for all outcomes, including ICH throughout the range between 2 and 12 MET-h/d (Figure 3B). For each outcome, the shape and strength of these associations with occupational and nonoccupational physical activity levels were broadly similar among men and women (eFigures 7-11 in the Supplement).

**Figure 4.**
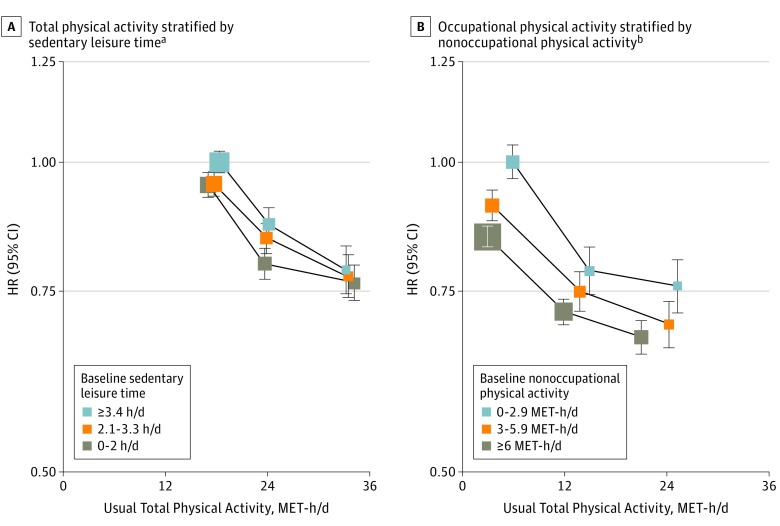
Adjusted Hazard Ratios (HRs) for Major Vascular Events and Occupational Physical Activity A, Major vascular events by total physical activity stratified by hours of sedentary leisure time. B, Occupational physical activity stratified by nonoccupational activity. Symbols and conventions as in Figure 1. MET-h/d indicates metabolic equivalent of task hours per day. ^a^All HRs are adjusted for age, sex, region, income, education, alcohol, smoking, fresh fruit intake, and self-rated general health status. ^b^For occupational physical activity, all HRs are adjusted for age, sex, region, income, education, alcohol, smoking, fresh fruit intake, sedentary leisure time, and self-rated general health status.

The associations of MVE with total physical activity levels were similar across different levels of sedentary leisure time (Figure 4A). Likewise, the inverse relationships of MVE with usual occupational physical activity levels were unaltered by different levels of nonoccupational physical activity (Figure 4B).

### Analyses by Participant Baseline Characteristics and by Further Adjustments or Exclusions

For each CVD outcome, the strength of the associations per 4 higher usual MET-h/d of physical activity was broadly similar between urban and rural areas and among subgroups that were classified by levels of alcohol consumption, smoking status, or self-reported health status (eFigures 12-14 in the Supplement). However, the effects appeared to be modified significantly by blood pressure and, to a lesser extent, by adiposity. Among people with a baseline systolic blood pressure of less than 140 mm Hg, 140 to 160 mm Hg, and more than160 mm Hg, the adjusted HRs for MVE per 4 higher usual MET-h/d were 0.91 (95% CI, 0.90-0.93), 0.97 (95% CI, 0.95-0.99), and 1.03 (95% CI, 1.01-1.05), respectively (*P* < .001), with similar patterns of association for each specific type of CVD (that were largely unaltered when using different clinical categorizations of systolic blood pressure, or when excluding those who were receiving blood pressure–lowering treatment). For MCE and CVD death, there was evidence of a somewhat stronger association among women and older participants (eFigures 13A and 14B in the Supplement).

Additional adjustments for other CVD risk factors (eg, adiposity, systolic blood pressure, dietary factors, lung function, and heart rate) attenuated the strength of the associations with MVE, as reflected by a decrease in the χ^2^ statistic. However, the inverse associations persisted throughout the ranges that were observed for these adjustments (eFigure 15 in the Supplement). The exclusion of participants with other prior nonvascular diseases, poor self-rated health, and CVD events that occurred during the first 3 years of follow-up had little effect on the overall observed associations (eTable 7 in the Supplement).

## Discussion

This large prospective study in China demonstrated that higher levels of physical activity had a strong inverse dose-response relationship with all major subtypes of CVD in both men and women. Throughout the range examined, a 4 MET-h/d higher level of physical activity (that could be obtained from either occupational or nonoccupational activities) was associated with highly statistically significant 5% to 12% lower risks of different subtypes of CVD. These effects were similar, and largely independent of each other, for both occupational and nonoccupational physical activity by different levels of sedentary leisure time, as reported in other studies.^12^ However, at higher levels of occupational physical activity (ie, >20 MET-h/d) the association with CVD events appeared to have flattened, especially for ICH.

A meta-analysis of 21 prospective studies that involved 20 000 CVD events reported that moderate levels of occupational physical activity were associated with an approximately 10% lower risk of both stroke and coronary heart disease in men, but with somewhat larger effects in women, especially for coronary heart disease.^11^ With almost twice the number of vascular events recorded as in that meta-analysis, this study demonstrated no major quantitative differences in the effects of physical activity levels on stroke between men and women, but with somewhat greater benefits for women for MCE and CVD death, consistent with previous findings in high-income populations.^11^ Findings from previous studies on stroke subtypes were inconclusive, with many studies having insufficient power to examine hemorrhagic stroke reliably,^34^ while a few other studies found no evidence of any differences between subtypes.^36^ Moreover, the range of physical activity levels examined in most previous studies was narrower than in this study, and tended to focus on fewer domains of physical activity.^35^ The present study demonstrated that while the inverse relationship with ischemic stroke was log-linear throughout the range of total physical activity that was examined, the association with ICH was log-linear up to levels of about 20 MET-h/d with an attenuation of the association thereafter. This attenuation was chiefly accounted for by occupational physical activity, suggesting that engaging in strenuous occupational activities (eg, approximately 6 MET) for more than 3 hours a day (ie, approximately 20 MET-h/d) may be somewhat less beneficial for ICH compared with those who engaged in more moderate levels of physical activity.

In a prospective study of UK women that involved 1800 ICH events,^39^ strenuous activity was associated with about a 20% lower risk of ICH for nondaily vs inactive but a nonsignificant 5% excess risk (95% CI, −33% to 17%) for daily strenuous activity vs inactive.^39^ In a recent Japanese cohort of 74 913 individuals with 1007 hemorrhagic stroke there was a J-shaped association of total physical activity (with a similar range to CKB) with hemorrhagic stroke.^40^ In the present study there was some evidence of increased risk of MVE, IS, and ICH among participants with a baseline systolic blood pressure of more than 160 mm Hg. In China, including those enrolled into CKB, fewer than 10% of individuals with hypertension had their blood pressure properly treated and controlled^41^ and vigorous physical activity in individuals with uncontrolled hypertension may be harmful.

The associations of higher physical activity levels and CVD events may be mediated partly through established CVD risk factors, including improved profiles of inflammation, adiposity, blood lipids, and blood pressure.^42^ Indeed, in this study, physical activity levels were weakly associated with adiposity and blood pressure and among a subset of the participants with blood lipids and markers of inflammation.

### Strengths and Limitations

This study’s chief strengths are the large sample size that permits the assessments of risk across a wider range of physical activity, information on both occupational and nonoccupational physical activity, and associations with both nonfatal and fatal vascular disease. Moreover, we were able to examine the associations, both shape and strength, across several important population subgroups such as by age, sex, adiposity, and blood pressure. However, the present study had several limitations, including self-reported physical activity only, no data that objectively quantified physical activity,^43^ and no data on cardiorespiratory fitness.^44^ Although the risk estimates for physical activity were corrected for regression dilution bias, we could not correct for measurement errors in covariates. Despite excluding the first 3 years of follow-up and individuals with prior diseases or poor health status, it is possible that reverse causality may still be present and residual confounding because of unknown, unmeasured factors (eg, lipids) or suboptimally measured factors (eg, dietary factors) cannot be completely discounted.

## Conclusions

This study provides new evidence that higher levels of total or domain-specific physical activity were inversely associated with risks of major cardiovascular diseases in Chinese adults. These associations appear to be proportional to the amount of physical activity that was undertaken within a broad range of physical activity typically seen in low- and middle-income countries, especially among those without hypertension.

## Notes

Supplement. **eTable 1: **Physical Activity Types, MET Values, Codes and Intensity Categories **eTable 2:** Mean (SD) Levels of Plasma Biochemical Markers by Levels of Physical Activity in a Subset of CKB Participants **eTable 3:** Types of Activity by Area and Gender **eTable 4: **Adjusted HRs for Major Cardiovascular Diseases by Total, Occupational and Nonoccupational Physical Activity **eTable 5: **Adjusted Event Rates for Major Cardiovascular Diseases by Baseline Levels of Total, Occupational and Nonoccupational Physical Activity **eTable 6:** Estimated Regression Dilution Ratio for Total, Occupational and Nonoccupational Physical Activity **eTable 7:** Adjusted HRs for Major Cardiovascular Diseases by Total, Occupational and Nonoccupational Physical Activity After Excluding Participants With Major Prior Chronic Diseases, Poor Self-Rated General Health and the First 3 Years of Follow-up **eFigure 1: **Location of the 10 areas Included in the China Kadoorie Biobank **eFigure 2:** Adjusted HRs for Major Vascular Events by Total Physical Activity, Stratified by A) Area and B) Gender **eFigure 3: **Adjusted HRs for Major Coronary Events by Total Physical Activity, Stratified by A) Area and B) Gender **eFigure 4:** Adjusted HRs for Ischemic Stroke Events by Total Physical Activity, Stratified by A) Area and B) Gender **eFigure 5:** Adjusted HRs for Intracerebral Hemorrhage Events by Total Physical Activity, Stratified by A) Area and B) Gender **eFigure 6:** Adjusted HRs for Cardiovascular Deaths by Total Physical Activity, Stratified by A) Area and b) Gender **eFigure 7:** Adjusted HRs for Major Vascular Events A) Occupational and B) Nonoccupational Physical Activity, in Men and Women **eFigure 8: **Adjusted HRs for Major Coronary Events A) Occupational and B) Nonoccupational Physical Activity, in Men and Women **eFigure 9:** Adjusted HRs for Ischaemic Stroke Events A) Occupational and B) Nonoccupational Physical Activity, in Men and Women **eFigure 10:** Adjusted HRs for Intracerebral Hemorrhage Events A) Occupational and B) Nonoccupational Physical Activity, in Men and Women **eFigure 11:** Adjusted HRs for Cardiovascular Deaths A) Occupational and B) Nonoccupational Physical Activity, in Men and Women **eFigure 12:** Adjusted HRs for Major Vascular Events per 4 MET-h/day Higher Usual Total Physical Activity by Baseline Characteristics **eFigure 13:** Adjusted HRs for A) Major Coronary Events and B) Ischemic Stroke per 4 MET-h/day Higher Usual Total Physical Activity by Baseline Characteristics **eFigure 14:** Adjusted HRs for A) Intracerebral Hemorrhage and B) Cardiovascular Death per 4 MET-h/day Higher Usual Total Physical Activity by Baseline Characteristics **eFigure 15:** Effect on HRs for Major Vascular Events per 4 MET-h/day Higher Usual Total Physical Activity After Sequential Adjustment for Potential Confounders
