# Supplementary material for: Association of Physical Activity With Risk of Major Cardiovascular Diseases in Chinese Men and Women
Source: JAMA Cardiol. 2017 Nov 8;2(12):1349–58. doi: 10.1001/jamacardio.2017.4069 (PMC5814992; doi:10.1001/jamacardio.2017.4069)
Supplement: Supplement. — eTable 1: Physical Activity Types, MET Values, Codes and Intensity Categories eTable 2: Mean (SD) Levels of Plasma Biochemical Markers by Levels of Physical Activity in a Subset of CKB Participants eTable 3: Types of Activity by Area and Gender eTable 4: Adjusted HRs for Major Cardiovascular Diseases by Total, Occupational and Nonoccupational Physical Activity eTable 5: Adjusted Event Rates for Major Cardiovascular Diseases by Baseline Levels of Total, Occupational and Nonoccupational Physical Activity eTable 6: Estimated Regression Dilution Ratio for Total, Occupational and Nonoccupational Physical Activity eTable 7: Adjusted HRs for Major Cardiovascular Diseases by Total, Occupational and Nonoccupational Physical Activity After Excluding Participants With Major Prior Chronic Diseases, Poor Self-Rated General Health and the First 3 Years of Follow-up eFigure 1: Location of the 10 areas Included in the China Kadoorie Biobank eFigure 2: Adjusted HRs for Major Vascular Events by Total Physical Activity, Stratified by A) Area and B) Gender eFigure 3: Adjusted HRs for Major Coronary Events by Total Physical Activity, Stratified by A) Area and B) Gender eFigure 4: Adjusted HRs for Ischemic Stroke Events by Total Physical Activity, Stratified by A) Area and B) Gender eFigure 5: Adjusted HRs for Intracerebral Hemorrhage Events by Total Physical Activity, Stratified by A) Area and B) Gender eFigure 6: Adjusted HRs for Cardiovascular Deaths by Total Physical Activity, Stratified by A) Area and b) Gender eFigure 7: Adjusted HRs for Major Vascular Events A) Occupational and B) Nonoccupational Physical Activity, in Men and Women eFigure 8: Adjusted HRs for Major Coronary Events A) Occupational and B) Nonoccupational Physical Activity, in Men and Women eFigure 9: Adjusted HRs for Ischaemic Stroke Events A) Occupational and B) Nonoccupational Physical Activity, in Men and Women eFigure 10: Adjusted HRs for Intracerebral Hemorrhage Events A) Occupational and B) Nonoccupational Phy [file jamacardiol-2-1349-s001.pdf]

Supplementary Online Content

Bennett D, Du H, Clarke RJ, et al; China Kadoorie Biobank Study Collaborative Group. Association of physical activity with risk of major cardiovascular diseases in Chinese men and women. *JAMA Cardiol*. Published online November 8, 2017. doi:10.1001/jamacardio.2017.4069

eTable 1: Physical Activity Types, MET Values, Codes and Intensity Categories

eTable 2: Mean (SD) Levels of Plasma Biochemical Markers by Levels of Physical Activity in a Subset of CKB Participants

eTable 3: Types of Activity by Area and Gender

eTable 4: Adjusted HRs for Major Cardiovascular Diseases by Total, Occupational and Nonoccupational Physical Activity

eTable 5: Adjusted Event Rates for Major Cardiovascular Diseases by Baseline Levels of Total, Occupational and Nonoccupational Physical Activity

eTable 6: Estimated Regression Dilution Ratio for Total, Occupational and Nonoccupational Physical Activity

eTable 7: Adjusted HRs for Major Cardiovascular Diseases by Total, Occupational and Nonoccupational Physical Activity After Excluding Participants With Major Prior Chronic Diseases, Poor Self-Rated General Health and the First 3 Years of Follow-up

eFigure 1: Location of the 10 areas Included in the China Kadoorie Biobank

eFigure 2: Adjusted HRs for Major Vascular Events by Total Physical Activity, Stratified by A) Area and B) Gender

eFigure 3: Adjusted HRs for Major Coronary Events by Total Physical Activity, Stratified by A) Area and B) Gender

eFigure 4: Adjusted HRs for Ischemic Stroke Events by Total Physical Activity, Stratified by A) Area and B) Gender

eFigure 5: Adjusted HRs for Intracerebral Hemorrhage Events by Total Physical Activity, Stratified by A) Area and B) Gender

eFigure 6: Adjusted HRs for Cardiovascular Deaths by Total Physical Activity, Stratified by A) Area and b) Gender

eFigure 7: Adjusted HRs for Major Vascular Events A) Occupational and B) Nonoccupational Physical Activity, in Men and Women

eFigure 8: Adjusted HRs for Major Coronary Events A) Occupational and B) Nonoccupational Physical Activity, in Men and Women

eFigure 9: Adjusted HRs for Ischaemic Stroke Events A) Occupational and B) Nonoccupational Physical Activity, in Men and Women

eFigure 10: Adjusted HRs for Intracerebral Hemorrhage Events A) Occupational and B) Nonoccupational Physical Activity, in Men and Women

© 2017 China Kadoorie Biobank Study Collaborative Group et al. *JAMA Cardiology.*

eFigure 11: Adjusted HRs for Cardiovascular Deaths A) Occupational and B) Nonoccupational Physical Activity, in Men and Women
eFigure 12: Adjusted HRs for Major Vascular Events per 4 MET-h/day Higher Usual Total Physical Activity by Baseline Characteristics
eFigure 13: Adjusted HRs for A) Major Coronary Events and B) Ischemic Stroke per 4 MET-h/day Higher Usual Total Physical Activity by Baseline Characteristics
eFigure 14: Adjusted HRs for A) Intracerebral Hemorrhage and B) Cardiovascular Death per 4 MET-h/day Higher Usual Total Physical Activity by Baseline Characteristics
eFigure 15: Effect on HRs for Major Vascular Events per 4 MET-h/day Higher Usual Total Physical Activity After Sequential Adjustment for Potential Confounders

This supplementary material has been provided by the authors to give readers additional information about their work.

© 2017 China Kadoorie Biobank Study Collaborative Group et al. *JAMA Cardiology.*

eTable 1: Physical activity types, MET values, codes and intensity categories*

Activity type	Intensity	MET	Codes*
Heavy manual work	Vigorous	6.5	11477
Manual work	Moderate	4.5	11476
Standing work	Moderate	3.8	Mean of 11610 and 11630
Sedentary work	Low	1.8	Mean of 11580, 11585, and 11590
Manual work in the farming season	Vigorous	6.3	Mean of 11145 and 11146
Semi-mechanized work in the farming season	Moderate	3.4	Mean of 11146 and 11147
Fully mechanized work in the farming season	Low	2.4	Mean of 11147 and 11170
Work outside the farming season	Low	2	11147
Walking	Moderate	4	17270
Bicycle	Vigorous	6.8	1011
Motorbike	Moderate	3.5	16030
Private or public transportation (such as bus, car, underground, and ferry)	Low	1.7	Mean of 16010, 16015, and 16016
Household activity	Low	2.8	Mean of 05030[†], 05040[†], 05035, 05055, 05070, 05090[†], 05092[†], 05184, 05197, and 05200
Tai-Chi/qigong/leisure walking	Moderate	3.3	Mean of 15670 and 17160
Jogging/aerobic exercise	Vigorous	7.4	Mean of 03015, 12020, and 12150
Ball games	Moderate	5.5	Mean of 15020[†], 15030[†], 15055, 15080, 15090, 15255, 15605[†], 15610[†], 15652, 15660, 15675, 15710[†], and 15711[†]
Brisk walking/gymnastics/folk dancing	Moderate	4.2	Mean of 03025, 15300, and 17200
Swimming	Vigorous	7.2	Mean of 18230, 18240, and 18310
Other exercise, e.g. mountain walking, home exercise and rope jumping	Moderate	5.9	Mean of 02010, 02064, 04001, 04100, 15110[†], 15120[†], 15200, 15240, 15310, 15425[†], 15430[†], 15537, 15550[‡], 15551[‡], 15552[‡], 15580, 15590, 15730, 15732[‡], 15733[‡], 15734[‡], and 19030

MET: Metabolic equivalent of tasks.
* Based on the 2011 Compendium of Physical Activities: a second update of codes and MET values. Ainsworth BE, et al. Medicine and Science in Sports and Exercise, 2011;43(8):1575-1581.
[†] Assigned 1/2 weight in calculating the mean MET value because the connecting two items represent one type of activity.
[‡] Assigned 1/3 weight in calculating the mean MET value because the connecting three items represent one type of activity.

© 2017 China Kadoorie Biobank Study Collaborative Group et al. *JAMA Cardiology.*

eTable 2: Mean (SD) levels of plasma biochemical markers by levels of physical activity in a subset of CKB participants

Biochemistry	No. of participants	Total physical activity (MET-h/day) ≤9.1	9.2 - 14.7	14.8 - 22.4	22.5 - 33.7	≥ 33.8	P-value for trend
Total cholesterol, mmol/L	9282	4.7 (0.9)	4.7 (0.9)	4.6 (0.9)	4.5 (0.9)	4.5 (0.9)	<0.0001
LDL-C, mmol/L	9282	2.4 (0.7)	2.4 (0.6)	2.3 (0.6)	2.2 (0.7)	2.3 (0.7)	<0.0001
HDL-C, mmol/L	9282	1.2 (0.3)	1.2 (0.3)	1.3 (0.3)	1.3 (0.3)	1.3 (0.3)	<0.0001
ApoB, mg/dl	9282	84.0 (20.4)	84.5 (19.5)	82.5 (19.1)	79.7 (19.8)	79.8 (20.3)	<0.0001
ApoA1, mg/dl	9282	133.6 (22.9)	134.7 (21.9)	136.2 (21.5)	137.7 (22.3)	138.0 (22.8)	<0.0001
Log triglycerides, log mmol/L	9282	0.6 (0.6)	0.6 (0.6)	0.5 (0.5)	0.5 (0.6)	0.4 (0.6)	<0.0001
Log Lp(a), log nmol/L	9282	3.0 (1.2)	3.0 (1.1)	3.0 (1.1)	3.0 (1.1)	3.0 (1.2)	0.7385
Fibrinogen, g/L	5247	3.1 (0.7)	3.1 (0.7)	3.1 (0.7)	3.1 (0.7)	3.1 (0.7)	0.0194
Log CRP, log mg/L	9282	0.2 (1.1)	0.1 (1.0)	-0.0 (1.0)	-0.0 (1.0)	-0.1 (1.0)	<0.0001
Log Gamma glutamyl tranferase, log u/L	8412	3.1 (0.7)	3.1 (0.7)	3.0 (0.6)	3.0 (0.7)	2.9 (0.7)	<0.0001
Albumin, g/L	8538	42.2 (2.8)	42.4 (2.7)	42.2 (2.6)	42.2 (2.7)	42.4 (2.8)	0.3488
Creatinine, umol/L	9282	65.5 (15.9)	64.5 (15.2)	62.8 (14.9)	63.1 (15.5)	61.8 (15.8)	<0.0001
Cystatin-C, mg/L	8117	0.9 (0.2)	0.8 (0.2)	0.8 (0.2)	0.8 (0.2)	0.8 (0.2)	<0.0001

All values are adjusted for age, sex, and area where appropriate.
To convert the values of total, LDL-C, HDL-C to milligrams per decilitre, divide by 0.0259.

© 2017 China Kadoorie Biobank Study Collaborative Group et al. *JAMA Cardiology.*

eTable 3: Types of activity by area and gender

	Rural Mean (SD)*		Urban Mean (SD)*		Total Mean (SD)	
	**Men (n = 113,983)**	**Women (n = 162,593)**	**Men (n = 85,035)**	**Women (n = 125,723)**	**Men (n =199,018)**	**Women (n = 288,316)**
**Total physical activity (MET-h/day)**	**24.8 (12.9)**	**22.2 (12.9)**	**20.4 (12.9)**	**18.5 (12.9)**	**22.9 (12.8)**	**20.6 (12.8)**
Non-occupational	5.3 (4.4)	10.3 (4.4)	5.9 (4.4)	9.6 (4.4)	5.5 (4.4)	10.0 (4.4)
Occupational	19.5 (12.5)	11.9 (12.5)	14.5 (12.5)	8.9 (12.5)	17.4 (12.4)	10.6 (12.4)
**Sedentary leisure-time, hours/day**	**3.0 (1.5)**	**2.9 (1.5)**	**3.2 (1.5)**	**3.0 (1.5)**	**3.1 (1.5)**	**2.9 (1.5)**

*All values were adjusted for age.

© 2017 China Kadoorie Biobank Study Collaborative Group et al. *JAMA Cardiology.*

eTable 4: Adjusted HRs for major cardiovascular diseases by total, occupational and non-occupational physical activity

Baseline physical activity (MET-h/day)	Major coronary events		Ischaemic stroke		Intracerebral haemorrhage		Cardiovascular deaths		Major vascular events	
	No. of events	HR* (95% CI)	No. of events	HR* (95% CI)	No. of events	HR* (95% CI)	No. of events	HR* (95% CI)	No. of events	HR* (95% CI)
**Total**										
≤9.1	2183	1.00 (0.95 - 1.05)	9364	1.00 (0.98 - 1.02)	1656	1.00 (0.95 - 1.06)	3611	1.00 (0.96 - 1.04)	13229	1.00 (0.98 - 1.02)
9.2 - 14.7	1197	0.83 (0.78 - 0.87)	6828	0.93 (0.91 - 0.95)	1185	0.86 (0.81 - 0.92)	1830	0.75 (0.72 - 0.79)	9177	0.90 (0.88 - 0.92)
14.8 - 22.4	702	0.74 (0.69 - 0.80)	4176	0.86 (0.84 - 0.89)	882	0.78 (0.73 - 0.83)	1206	0.67 (0.63 - 0.71)	5846	0.83 (0.81 - 0.86)
22.5 - 33.7	586	0.71 (0.65 - 0.78)	2929	0.83 (0.80 - 0.86)	827	0.70 (0.65 - 0.75)	1061	0.60 (0.56 - 0.64)	4408	0.79 (0.76 - 0.81)
≥ 33.8	414	0.69 (0.62 - 0.77)	2350	0.79 (0.75 - 0.82)	702	0.77 (0.71 - 0.83)	729	0.59 (0.55 - 0.64)	3524	0.77 (0.74 - 0.80)
P for non-linearity		<0.0001		0.0004		0.0001		<0.0001		<0.0001
**Occupational**†										
0	2743	1.00 (0.94 - 1.07)	13352	1.00 (0.97 - 1.03)	1954	1.00 (0.94 - 1.07)	4164	1.00 (0.95 - 1.05)	18020	1.00 (0.98 - 1.03)
0.1 - 5.9	648	0.77 (0.70 - 0.84)	3467	0.89 (0.86 - 0.93)	871	0.87 (0.81 - 0.94)	1276	0.75 (0.70 - 0.80)	5012	0.87 (0.84 - 0.90)
6.0 - 13.8	698	0.70 (0.65 - 0.76)	3577	0.83 (0.81 - 0.86)	949	0.75 (0.71 - 0.80)	1260	0.66 (0.62 - 0.69)	5312	0.80 (0.78 - 0.82)
13.9 - 25.7	548	0.69 (0.63 - 0.76)	2713	0.82 (0.79 - 0.85)	833	0.70 (0.65 - 0.75)	1054	0.61 (0.58 - 0.66)	4150	0.77 (0.75 - 0.80)
≥ 25.8	445	0.68 (0.62 - 0.76)	2538	0.79 (0.75 - 0.82)	645	0.75 (0.69 - 0.82)	683	0.59 (0.55 - 0.65)	3690	0.77 (0.74 - 0.79)
P for non-linearity		<0.0001		0.54		0.0001		<0.0001		<0.0001
**Non-occupational**†										
0 - 3.9	1273	1.00 (0.94 - 1.07)	5358	1.00 (0.97 - 1.03)	1139	1.00 (0.93 - 1.07)	2047	1.00 (0.95 - 1.05)	7801	1.00 (0.97 - 1.03)
4.0 - 6.5	1020	0.88 (0.82 - 0.93)	4921	0.94 (0.91 - 0.96)	977	0.91 (0.85 - 0.97)	1685	0.89 (0.85 - 0.93)	6992	0.92 (0.90 - 0.95)
6.6 - 8.4	1027	0.86 (0.81 - 0.91)	5211	0.93 (0.90 - 0.95)	1089	0.89 (0.84 - 0.95)	1795	0.85 (0.81 - 0.89)	7369	0.90 (0.88 - 0.93)
8.5 - 11.6	881	0.80 (0.75 - 0.85)	4930	0.90 (0.88 - 0.93)	1025	0.89 (0.84 - 0.95)	1485	0.78 (0.74 - 0.82)	6876	0.88 (0.86 - 0.90)
≥ 11.7	881	0.78 (0.72 - 0.84)	5227	0.88 (0.85 - 0.90)	1022	0.84 (0.78 - 0.90)	1425	0.71 (0.67 - 0.75)	7146	0.85 (0.83 - 0.87)
P for non-linearity		0.41		0.99		0.62		0.77		

* All HRs are adjusted for stratified by age at risk, sex, and region and adjusted for income, education, BMI, alcohol, smoking, SBP, fresh fruit intake, sedentary leisure time, and self-rated health.
† Occupational physical activity additionally adjusted for non-occupational physical activity. Non-occupational physical activity additionally adjusted for occupational physical activity.

© 2017 China Kadoorie Biobank Study Collaborative Group et al. *JAMA Cardiology.*

eTable 5: Adjusted event rates for major cardiovascular diseases by baseline levels of total, occupational and non-occupational physical activity

Baseline physical activity (MET-h/day)	Major coronary events		Ischaemic stroke		Intracerebral haemorrhage		Cardiovascular deaths		Major vascular events	
	No. of events	Event rate* (95% CI)	No. of events	Event rate* (95% CI)	No. of events	Event rate* (95% CI)	No. of events	Event rate* (95% CI)	No. of events	Event rate* (95% CI)
**Total**										
≤ 9.1	2183	1.66 (1.56 - 1.76)	9364	7.53 (7.33 - 7.73)	1656	1.87 (1.72 - 2.01)	3611	3.12 (2.96 - 3.28)	13229	11.35 (11.1 -11.63)
9.2 - 14.7	1197	1.32 (1.22 - 1.41)	6828	6.92 (6.73 - 7.11)	1185	1.46 (1.35 - 1.56)	1830	2.10 (1.98 - 2.22)	9177	9.74 (9.50 - 9.98)
14.8 - 22.4	702	1.11 (1.02 - 1.20)	4176	6.49 (6.27 - 6.71)	882	1.27 (1.17 - 1.36)	1206	1.84 (1.72 - 1.96)	5846	8.99 (8.73 - 9.25)
22.5 - 33.7	586	1.11 (0.91 - 1.31)	2929	6.49 (6.07 - 6.92)	827	1.18 (1.03 - 1.34)	1061	1.63 (1.42 - 1.84)	4408	8.91 (8.41 - 9.41)
≥ 33.8	414	0.96 (0.75 - 1.17)	2350	5.88 (5.31 - 6.46)	702	1.37 (1.16 - 1.57)	729	1.69 (1.39 - 2.00)	3524	8.42 (7.74 - 9.09)
**Occupational**										
0	2743	1.67 (1.54 - 1.80)	13352	7.41 (7.18 - 7.64)	1954	1.89 (1.70 - 2.08)	4164	3.13 (2.93 - 3.33)	18371	11.50 (11.1 -11.85)
0.1 - 5.9	648	0.91 (0.76 - 1.06)	3467	6.95 (6.06 - 7.84)	871	1.45 (1.25 - 1.66)	1276	1.91 (1.71 - 2.12)	5085	9.79 (8.85 -10.73)
6.0 - 13.8	698	0.95 (0.86 - 1.04)	3577	6.06 (5.70 - 6.41)	949	1.22 (1.11 - 1.32)	1260	1.73 (1.51 - 1.95)	5425	8.59 (8.16 - 9.02)
13.9 - 25.7	548	1.20 (0.76 - 1.65)	2713	7.29 (6.36 - 8.22)	833	1.26 (1.00 - 1.52)	1054	1.79 (1.34 - 2.24)	4230	9.93 (8.90 -10.97)
≥ 25.8	445	1.22 (0.83 - 1.61)	2538	6.02 (5.45 - 6.58)	645	1.43 (1.07 - 1.78)	683	2.20 (1.50 - 2.91)	3771	9.20 (8.28 -10.12)
**Non-occupational**										
0 - 3.9	1273	1.65 (1.50 - 1.80)	5358	7.72 (7.33 - 8.11)	1139	1.93 (1.64 - 2.22)	2047	3.30 (2.95 - 3.65)	7964	11.81 (11.3 -12.35)
4.0 - 6.5	1020	1.40 (1.29 - 1.50)	4921	6.96 (6.73 - 7.18)	977	1.39 (1.27 - 1.50)	1685	2.39 (2.25 - 2.53)	7132	10.11 (9.83 -10.39)
6.6 - 8.4	1027	1.29 (1.21 - 1.38)	5211	6.83 (6.63 - 7.03)	1089	1.45 (1.36 - 1.55)	1795	2.28 (2.16 - 2.40)	7490	9.83 (9.58 -10.07)
8.5 - 11.6	881	1.23 (1.14 - 1.32)	4930	6.59 (6.39 - 6.80)	1025	1.39 (1.29 - 1.49)	1485	2.03 (1.91 - 2.14)	7013	9.49 (9.24 - 9.74)
≥ 11.7	881	1.21 (1.09 - 1.33)	5227	6.48 (6.23 - 6.73)	1022	1.26 (1.14 - 1.37)	1425	1.92 (1.76 - 2.07)	7283	9.24 (8.92 - 9.55)

*All event rates are per 1,000 person-years and are adjusted for age, sex, and area.

© 2017 China Kadoorie Biobank Study Collaborative Group et al. *JAMA Cardiology.*

eTable 6: Estimated regression dilution ratio for total, occupational and non-occupational physical activity

Baseline physical activity groups	Mean Baseline Physical activity (MET-h/day)	Mean First Re-survey Physical activity (MET-h/day)	Ratio of the ranges (MacMahon-Peto) regression dilution ratio estimate	Self-correlation regression dilution ratio estimate
**Total**				
≤9.1 (i)	5.95	12.02		
9.2 -14.7 (ii)	11.97	15.09		
14.8 - 22.4 (iii)	18.23	19.34	0.53	0.51
22.5 - 33.7 (iv)	27.85	25.44		
≥33.8 (v)	43.58	31.98		
Difference (v–i)	***37.63***	***19.96***		
**Occupational**				
0 (i)	0	2.43		
0.1 - 5.9 (ii)	3.15	8.73		
6.0 - 13.8 (iii)	9.97	12.37	0.63	0.57
13.9 - 25.7 (iv)	18.85	17.95		
≥ 25.8 (iv)	35.71	25.08		
Difference (v–i)	***35.71***	***22.65***		
**Non-occupational**				
0 - 3.9 (i)	2.11	4.58		
4.0 - 6.5 (ii)	5.30	6.74		
6.6 - 8.4 (iii)	7.70	8.24	0.50	0.48
8.5 - 11.6 (iv)	10.08	9.20		
≥11.7 (v)	15.53	11.15		
Difference (v–i)	***13.42***	***6.57***		

© 2017 China Kadoorie Biobank Study Collaborative Group et al. *JAMA Cardiology.*

eTable 7: Adjusted HRs* for major cardiovascular diseases by total, occupational and non-occupational physical activity after excluding participants with major prior chronic diseases†, poor self-rated general health and the first three years of follow-up

	Major coronary events		Ischaemic stroke		Intracerebral haemorrhage		Cardiovascular deaths		Major vascular events	
**Baseline physical activity (MET-h/day)**	**No. of events**	**HR* (95% CI)**	**No. of events**	**HR* (95% CI)**	**No. of events**	**HR* (95% CI)**	**No. of events**	**HR* (95% CI)**	**No. of events**	**HR* (95% CI)**
**Total**										
≤9.1	1334	1.00 (0.94 - 1.07)	6679	1.00 (0.97 - 1.03)	964	1.00 (0.93 - 1.08)	1985	1.00 (0.95 - 1.05)	8876	1.00 (0.98 - 1.02)
9.2 - 14.7	819	0.87 (0.81 - 0.93)	5068	0.91 (0.89 - 0.94)	845	0.95 (0.89 - 1.02)	1180	0.80 (0.76 - 0.85)	6683	0.91 (0.89 - 0.93)
14.8 - 22.4	499	0.82 (0.75 - 0.89)	3213	0.86 (0.83 - 0.89)	609	0.83 (0.76 - 0.89)	785	0.73 (0.68 - 0.79)	4355	0.85 (0.83 - 0.88)
22.5 - 33.7	413	0.76 (0.68 - 0.85)	2273	0.81 (0.78 - 0.85)	599	0.76 (0.69 - 0.83)	708	0.64 (0.59 - 0.69)	3314	0.79 (0.76 - 0.82)
≥33.8	317	0.78 (0.69 - 0.88)	1880	0.77 (0.74 - 0.81)	537	0.84 (0.77 - 0.93)	525	0.66 (0.60 - 0.73)	2777	0.79 (0.76 - 0.82)
P for non-linearity		0.064		0.0004		<0.0001		<0.0001		0.0005
**Occupational**										
0	1754	1.00 (0.92 - 1.09)	9595	1.00 (0.97 - 1.04)	1201	1.00 (0.92 - 1.09)	2406	1.00 (0.94 - 1.07)	12409	1.00 (0.97 - 1.03)
0.1 - 5.9	395	0.77 (0.68 - 0.86)	2583	0.88 (0.84 - 0.92)	587	0.93 (0.85 - 1.02)	751	0.76 (0.70 - 0.83)	3553	0.87 (0.84 - 0.91)
6.0 - 13.8	489	0.76 (0.70 - 0.83)	2742	0.81 (0.78 - 0.85)	646	0.79 (0.73 - 0.85)	788	0.69 (0.64 - 0.74)	3916	0.81 (0.78 - 0.83)
13.9 - 25.7	396	0.75 (0.67 - 0.83)	2146	0.81 (0.77 - 0.85)	618	0.76 (0.69 - 0.82)	740	0.67 (0.62 - 0.73)	3181	0.79 (0.76 - 0.82)
≥25.8	348	0.75 (0.67 - 0.84)	2047	0.77 (0.73 - 0.81)	502	0.82 (0.74 - 0.90)	498	0.66 (0.60 - 0.73)	2946	0.78 (0.75 - 0.81)
P for non-linearity		0.0036		0.0002		0.0043		0.0002		<0.0001
**Non-occupational**										
0 - 3.9	814	1.00 (0.92 - 1.08)	3969	1.00 (0.96 - 1.04)	717	1.00 (0.92 - 1.09)	1170	1.00 (0.93 - 1.07)	5459	1.00 (0.97 - 1.03)
4.0 - 6.5	689	0.90 (0.83 - 0.97)	3692	0.93 (0.90 - 0.96)	670	0.97 (0.90 - 1.05)	1034	0.92 (0.86 - 0.98)	5064	0.93 (0.91 - 0.96)
6.6 - 8.4	674	0.88 (0.81 - 0.95)	3885	0.90 (0.87 - 0.93)	738	0.95 (0.89 - 1.03)	1106	0.89 (0.84 - 0.94)	5286	0.90 (0.88 - 0.93)
8.5 - 11.6	593	0.81 (0.74 - 0.88)	3689	0.87 (0.84 - 0.90)	720	0.96 (0.89 - 1.03)	943	0.80 (0.75 - 0.86)	5021	0.88 (0.86 - 0.91)
≥11.7	612	0.82 (0.75 - 0.89)	3878	0.84 (0.81 - 0.87)	709	0.89 (0.82 - 0.98)	930	0.72 (0.67 - 0.78)	5175	0.84 (0.82 - 0.87)
P for non-linearity		0.50		0.49		0.80		0.28		0.75

* All HRs are stratified by age at risk, sex, and region and adjusted for income, education, alcohol, smoking, fresh fruit intake, sedentary leisure-time and mutually adjusted for other domain-specific physical activity when appropriate.

† Exclusions include prior history of CVD, tuberculosis, chronic obstructive pulmonary disease, emphysema or bronchitis, asthma, rheumatic heart disease and cancer.

© 2017 China Kadoorie Biobank Study Collaborative Group et al. *JAMA Cardiology.*

eFigure 1: The location of the 10 areas included in the China Kadoorie Biobank

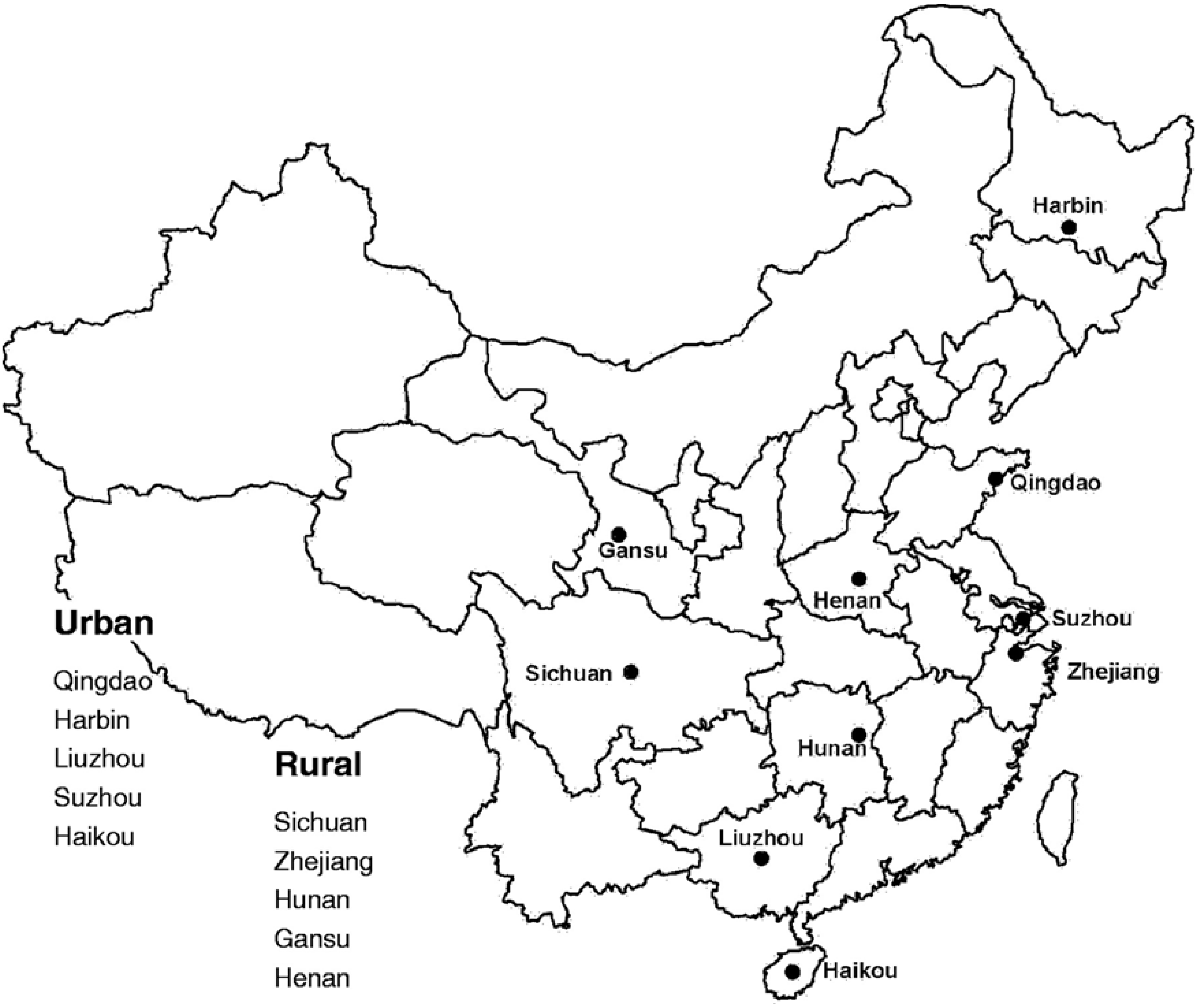

© 2017 China Kadoorie Biobank Study Collaborative Group et al. *JAMA Cardiology.*

eFigure 2: Adjusted HRs for major vascular events by total physical activity stratified by a) area and b) gender

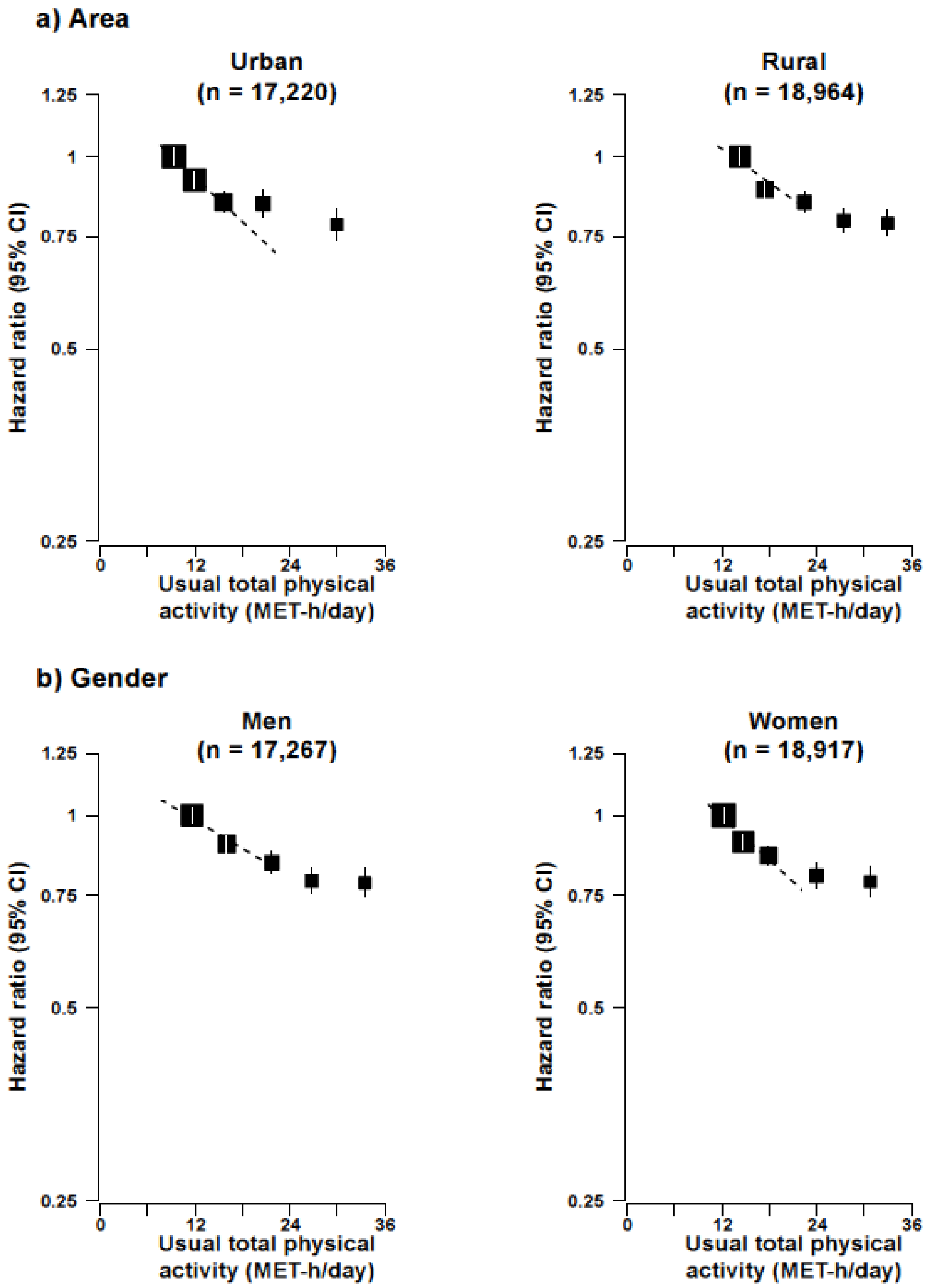

© 2017 China Kadoorie Biobank Study Collaborative Group et al. *JAMA Cardiology.*

eFigure 3: Adjusted HRs for major coronary events by total physical activity, stratifed by a) area and b) gender

a) Area

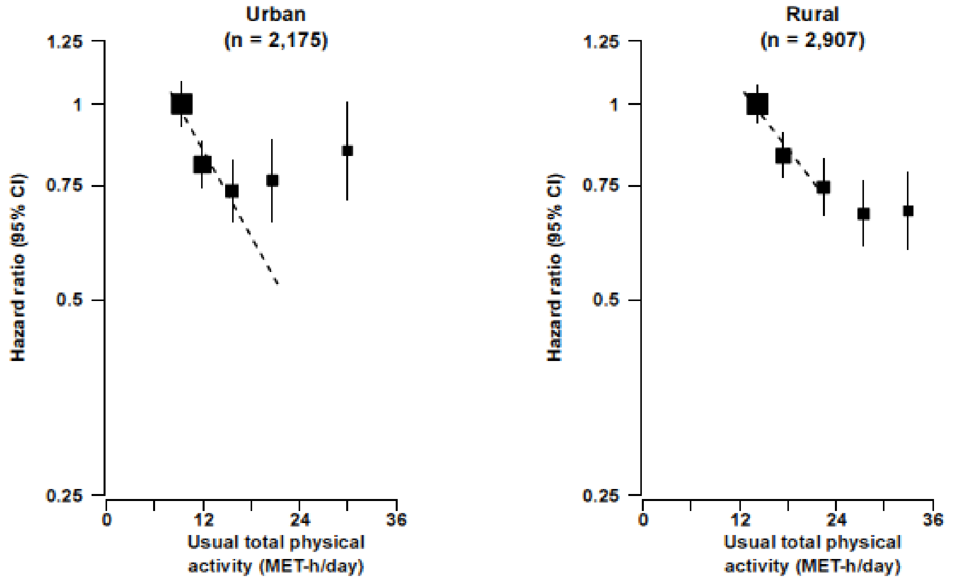

b) Gender

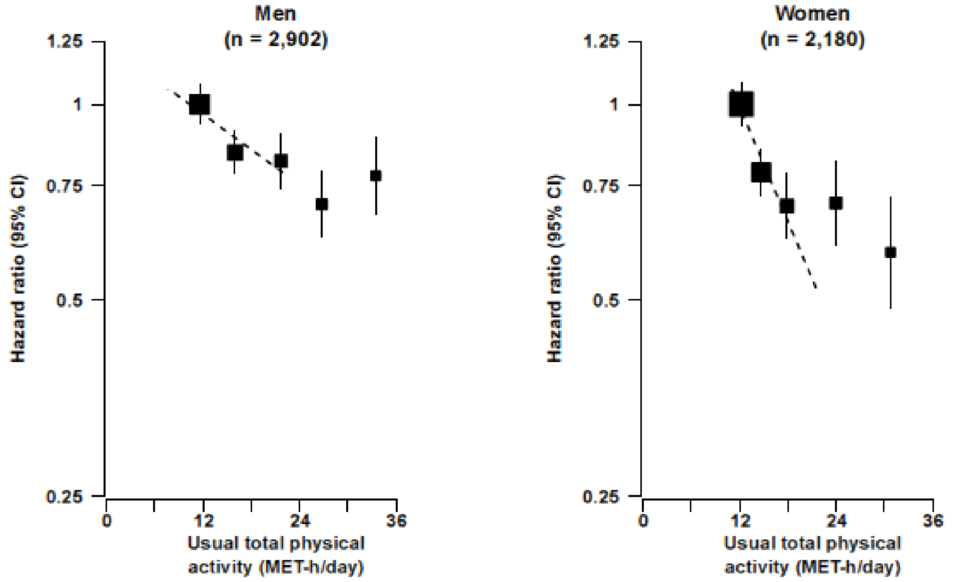

© 2017 China Kadoorie Biobank Study Collaborative Group et al. *JAMA Cardiology.*

eFigure 4: Adjusted HRs for ischemic stroke events by total physical activity, stratified by a) area and b) gender

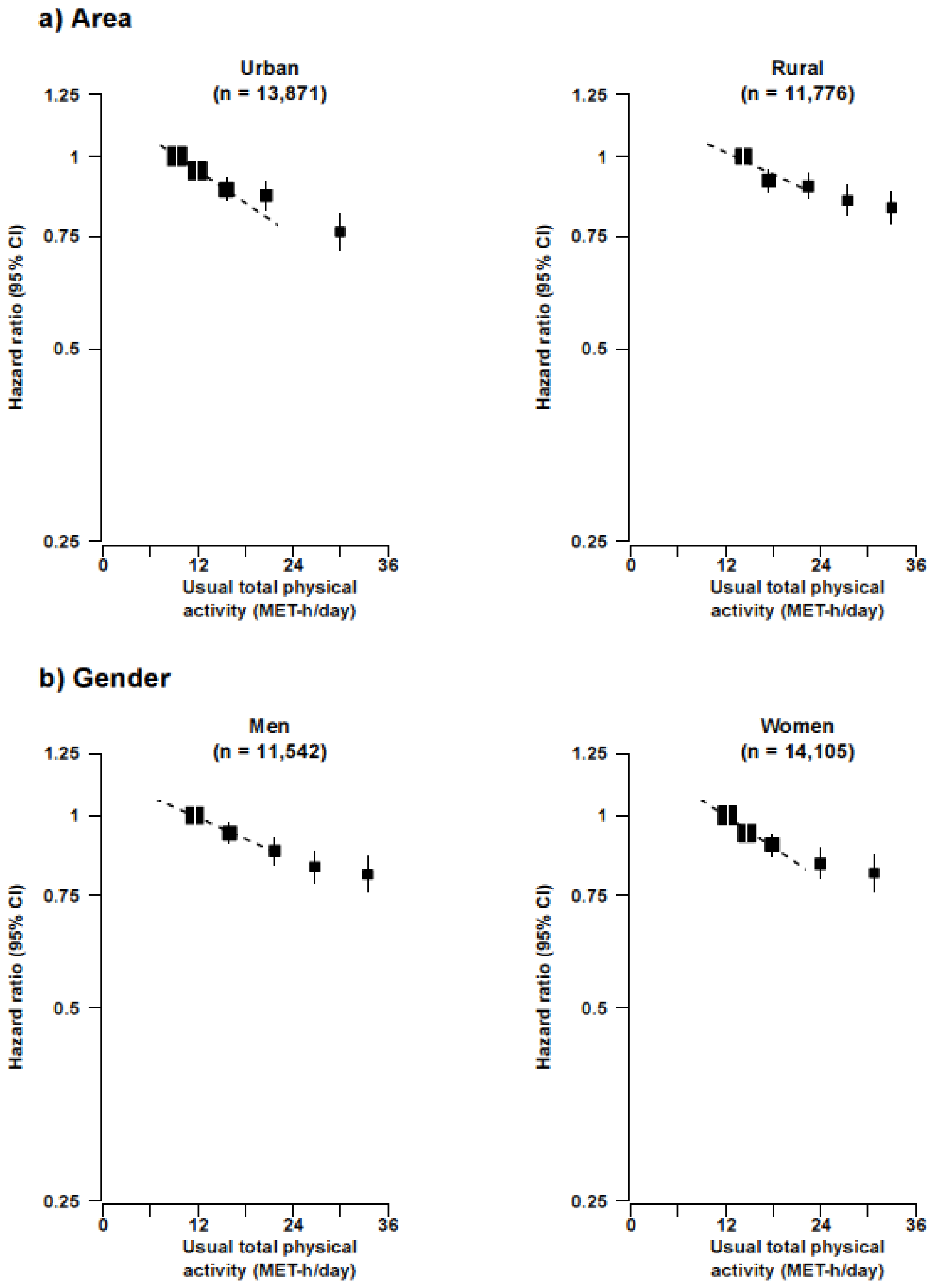

© 2017 China Kadoorie Biobank Study Collaborative Group et al. *JAMA Cardiology.*

eFigure 5: Adjusted HRs for intracerebral haemorrhage by total physical activity, stratified by a) area and b) gender

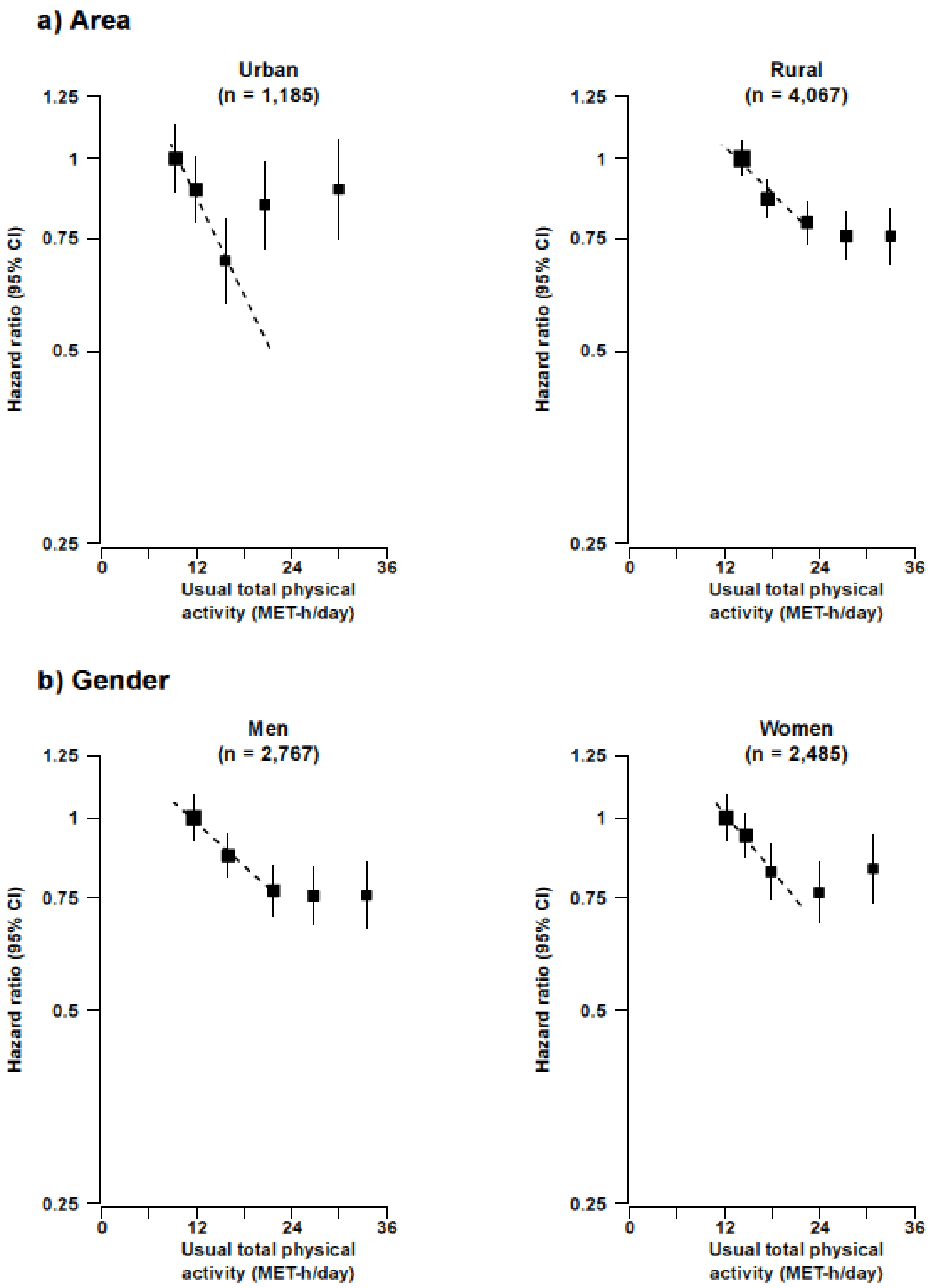

© 2017 China Kadoorie Biobank Study Collaborative Group et al. *JAMA Cardiology.*

eFigure 6: Adjusted HRs for cardiovascular death by total physical activity, stratified by a) area and b) gender

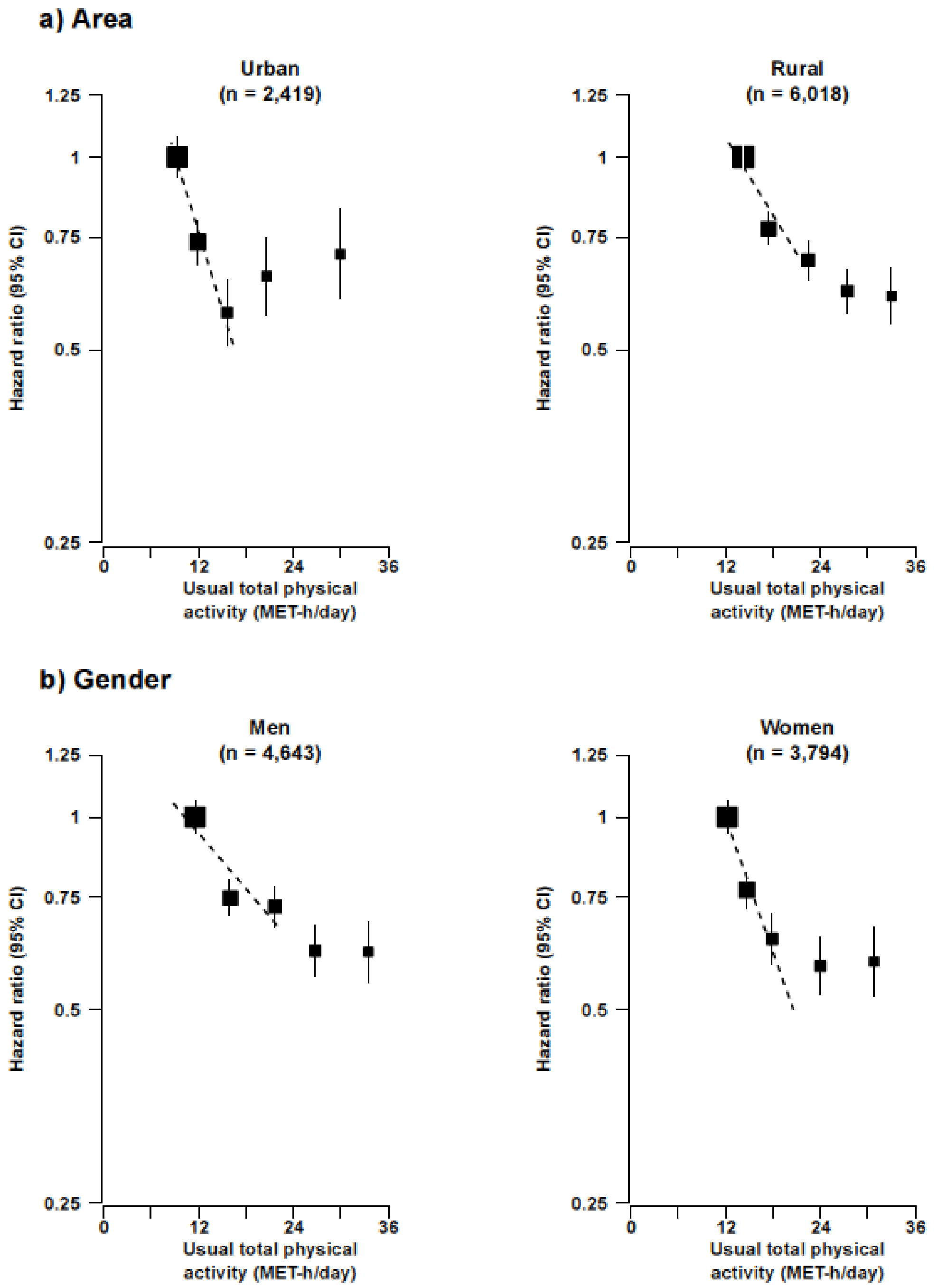

© 2017 China Kadoorie Biobank Study Collaborative Group et al. *JAMA Cardiology.*

eFigure 7: Adjusted HRs for major vascular events by a) occupational and b) non-occupational physical activity, in men and women

a) Occupational physical activity

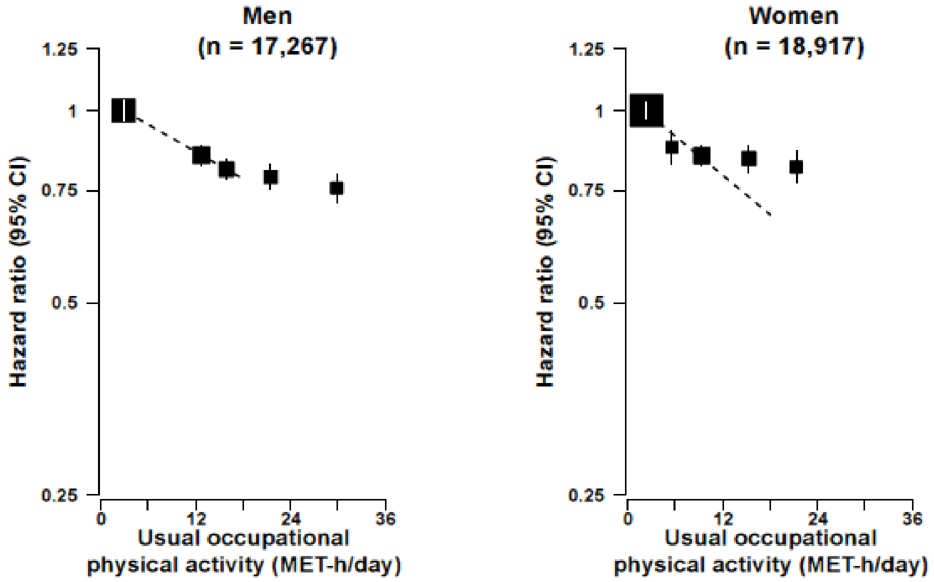

b) Non-occupational physical activity

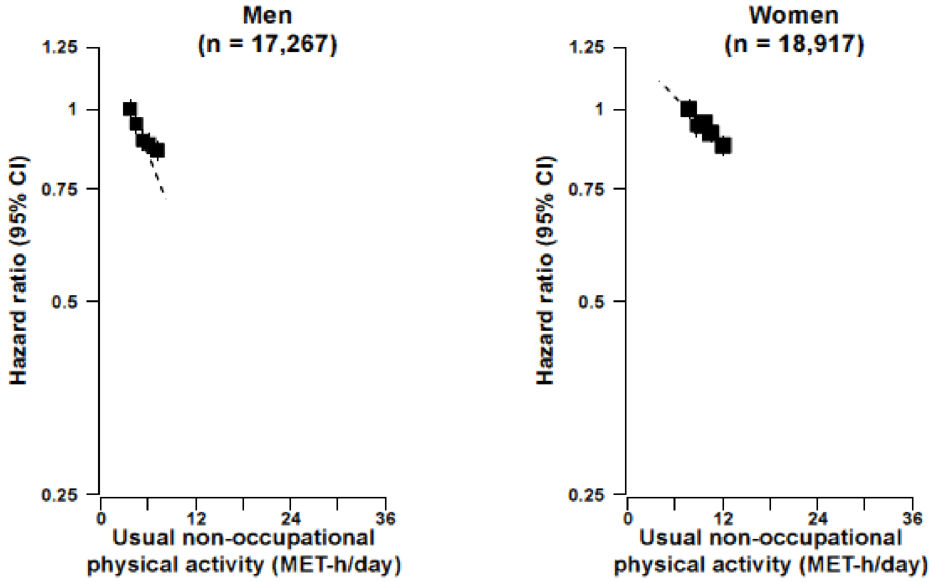

© 2017 China Kadoorie Biobank Study Collaborative Group et al. *JAMA Cardiology.*

eFigure 8: Adjusted HRs for major coronary events by a) occupational and b) non-occupational physical activity, in men and women

a) Occupational physical activity

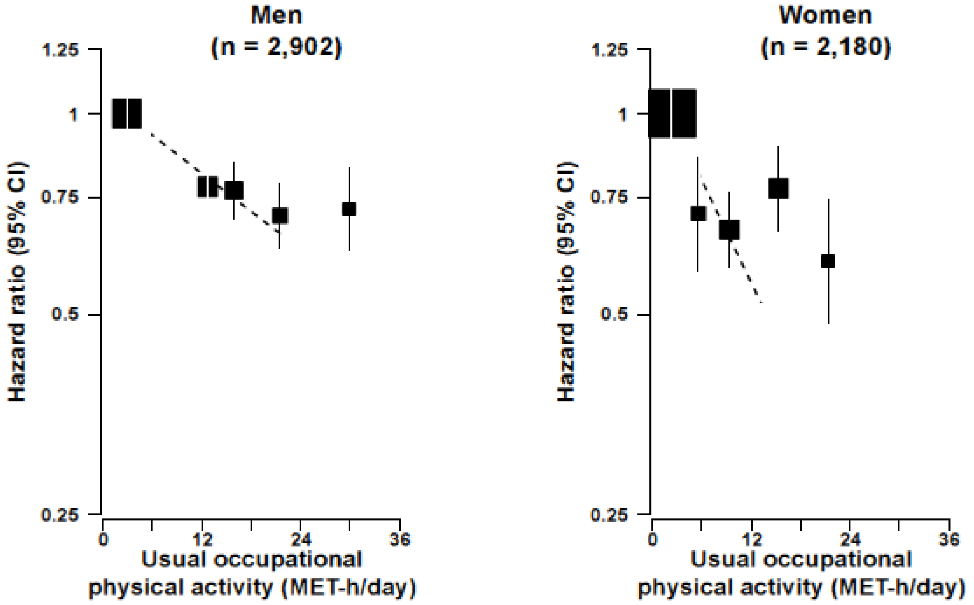

b) Non-occupational physical activity

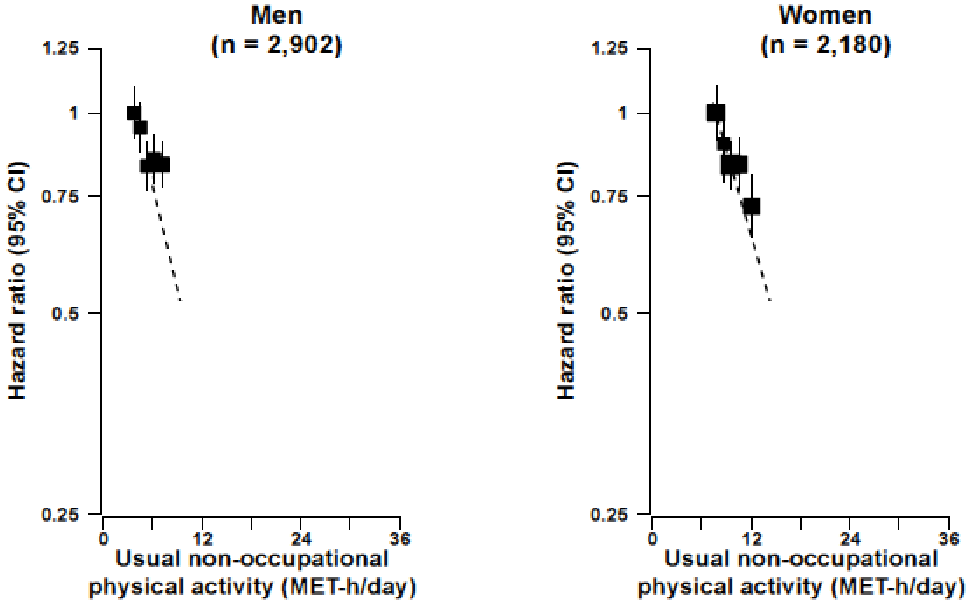

© 2017 China Kadoorie Biobank Study Collaborative Group et al. *JAMA Cardiology.*

eFigure 9: Adjusted HRs for ischaemic stroke by a) occupational and b) non-occupational physical activity, in men and women

a) Occupational physical activity

b) Non-occupational physical activity

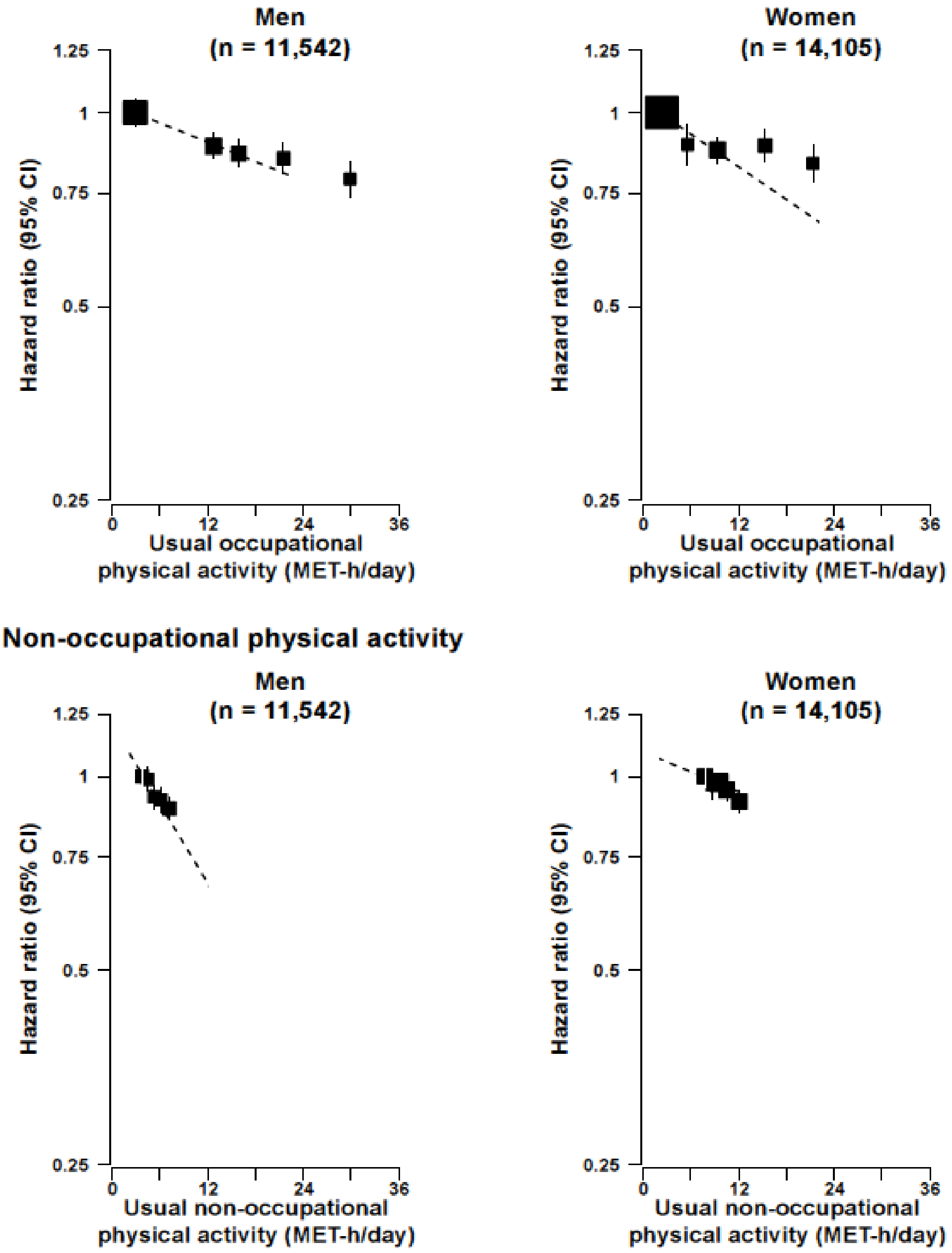

© 2017 China Kadoorie Biobank Study Collaborative Group et al. *JAMA Cardiology.*

eFigure 10: Adjusted HRs for intracerebral haemorrhage by a) occupational and b) non-occupational physical activity, in men and women

a) Occupational physical activity

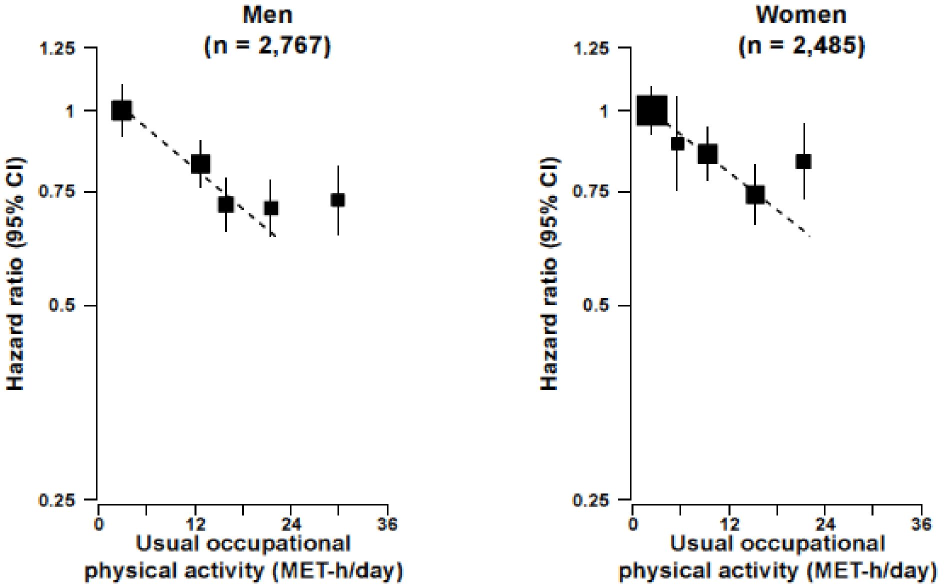

b) Non-occupational physical activity

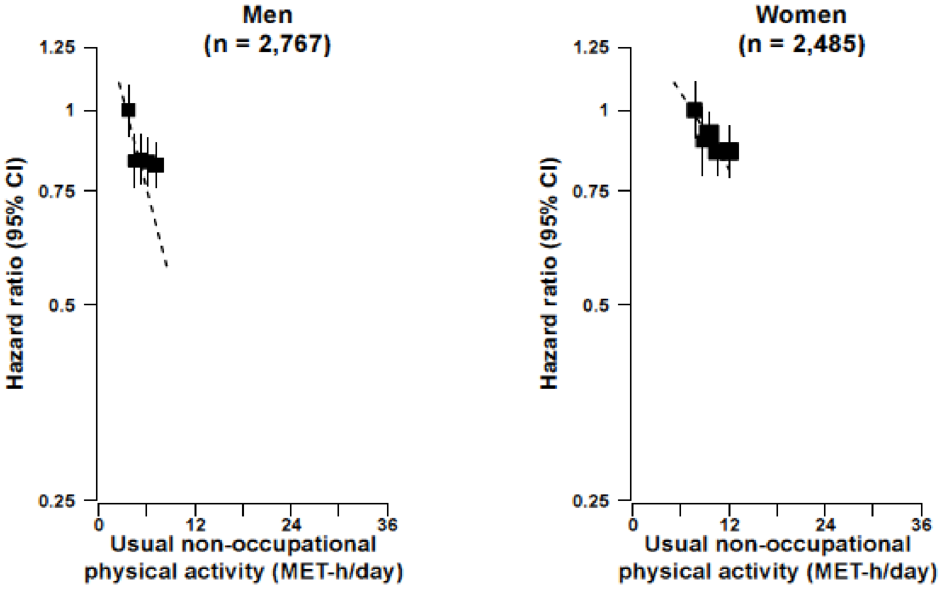

© 2017 China Kadoorie Biobank Study Collaborative Group et al. *JAMA Cardiology.*

eFigure 11: Adjusted HRs for fatal cardiovascular disease by a) occupational anc b) non-occupational physical activity, in men and women

a) Occupational physical activity

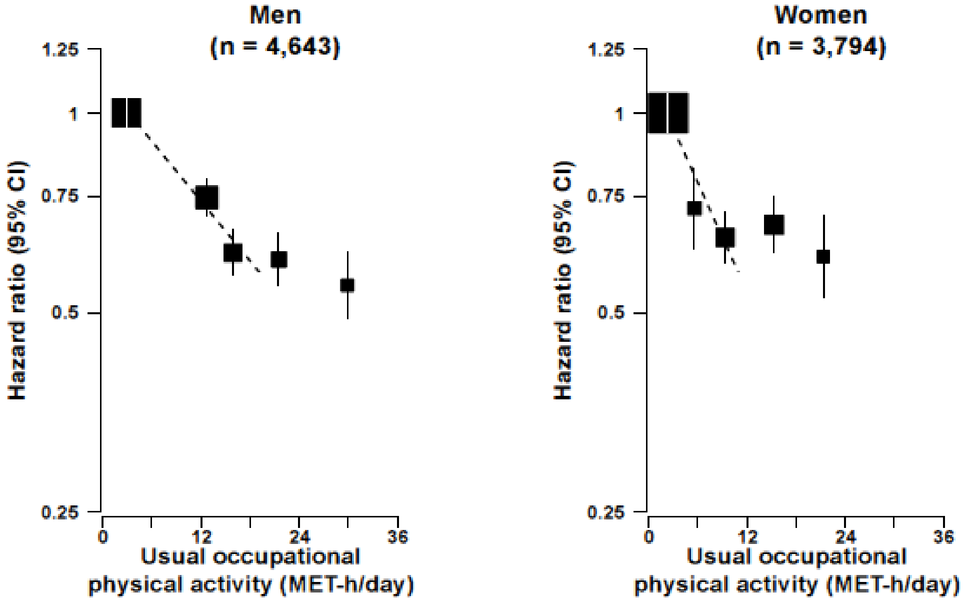

b) Non-occupational physical activity

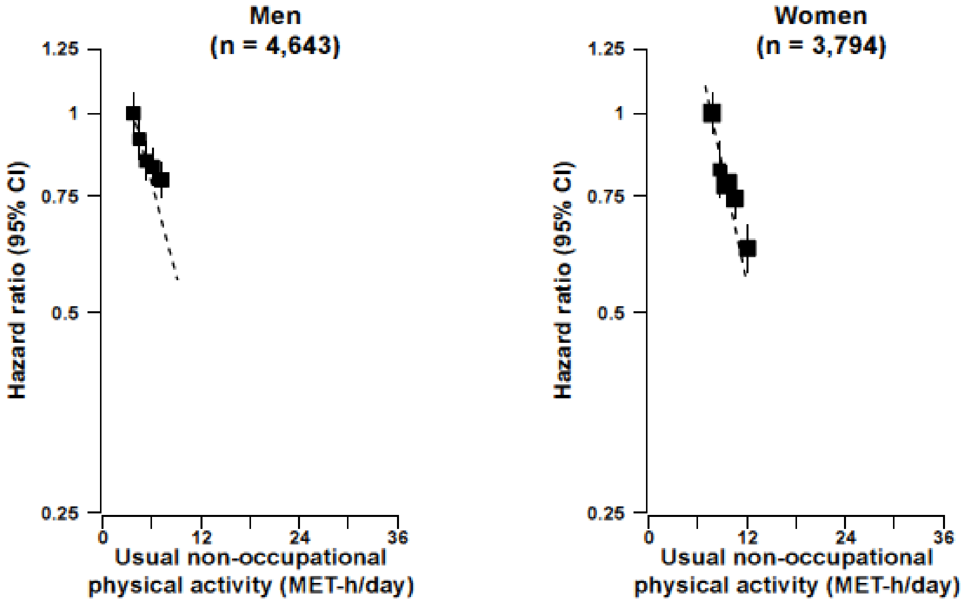

© 2017 China Kadoorie Biobank Study Collaborative Group et al. *JAMA Cardiology.*

eFigure 12: Adjusted HRs for major vascular events per 4 MET–h/day higher us[illegible] total physical activity by baseline characteristics

Subgroup	No. of cases	Mean (SD) MET–h/day	Hazard Ratio per 4 usual MET-h/day increase (99% CI)	χ^2_1 (P for trend or heterogeneity
**Age at risk, years**				
35 – 59	11666	20.76 (13.44)	0.96 (0.94,0.97)	6.2 (1.36E–02)
60 – 69	12180	15.26 (11.52)	0.95 (0.93,0.96)	
70 – 79	12338	10.82 (8.51)	0.93 (0.91,0.95)	
**Sex**				
Male	17267	16.11 (13.74)	0.95 (0.94,0.96)	0.7 (3.95E–01)
Female	18917	14.98 (10.13)	0.94 (0.93,0.96)	
**Region**				
Urban	17220	13.17 (10.07)	0.95 (0.94,0.97)	0.8 (3.78E–01)
Rural	18964	17.65 (13.16)	0.95 (0.93,0.96)	
**Alcohol**				
Never	16256	15.01 (11.09)	0.94 (0.92,0.95)	7.7 (9.59E–03)
Ex–regular	1064	13.18 (11.34)	0.93 (0.88,0.98)	
Current Regular	18864	16.09 (12.73)	0.96 (0.94,0.97)	
**Smoking**				
Never	20037	14.89 (10.49)	0.94 (0.93,0.96)	1.5 (2.45E–01)
Ex–regular/Occasional	5304	14.40 (12.55)	0.95 (0.92,0.97)	
Current Regular	10843	17.23 (14.00)	0.95 (0.94,0.97)	
**Body Mass Index, kg/ m^2**				
< 23	14327	16.18 (12.33)	0.94 (0.92,0.95)	17.0 (1.08E–04)
23 – 27	14773	15.48 (12.07)	0.96 (0.94,0.97)	
> 27	7084	14.27 (11.04)	0.97 (0.95,1.00)	
**Systolic Blood Pressure, mmHg**				
< 140	16979	15.84 (12.01)	0.91 (0.90,0.93)	195.5 (4.39E–39)
140 – 160	10317	15.13 (12.00)	0.97 (0.95,0.99)	
> 160	8888	15.35 (11.97)	1.03 (1.01,1.05)	
**Occupation**				
Farmer	13468	20.32 (13.19)	0.96 (0.95,0.98)	9.0 (3.79E–03)
Non–farmer	22716	12.67 (10.22)	0.94 (0.93,0.96)	
**Diabetes***				
No	33573	15.80 (12.14)	0.95 (0.94,0.96)	8.6 (3.92E–03)
Yes	2611	11.92 (9.36)	1.00 (0.96,1.05)	
**Hypertension***				
No	27606	15.92 (12.29)	0.94 (0.93,0.95)	91.0 (3.41E–19)
Yes	8578	14.24 (10.93)	1.02 (1.00,1.04)	
**General health***				
Good	31109	15.83 (12.11)	0.96 (0.95,0.97)	5.6 (2.34E–02)
Poor	5075	13.63 (11.15)	0.93 (0.91,0.96)	
**All**	**36184**	**15.61 (12.02)**	**0.94 (0.93,0.95)**	

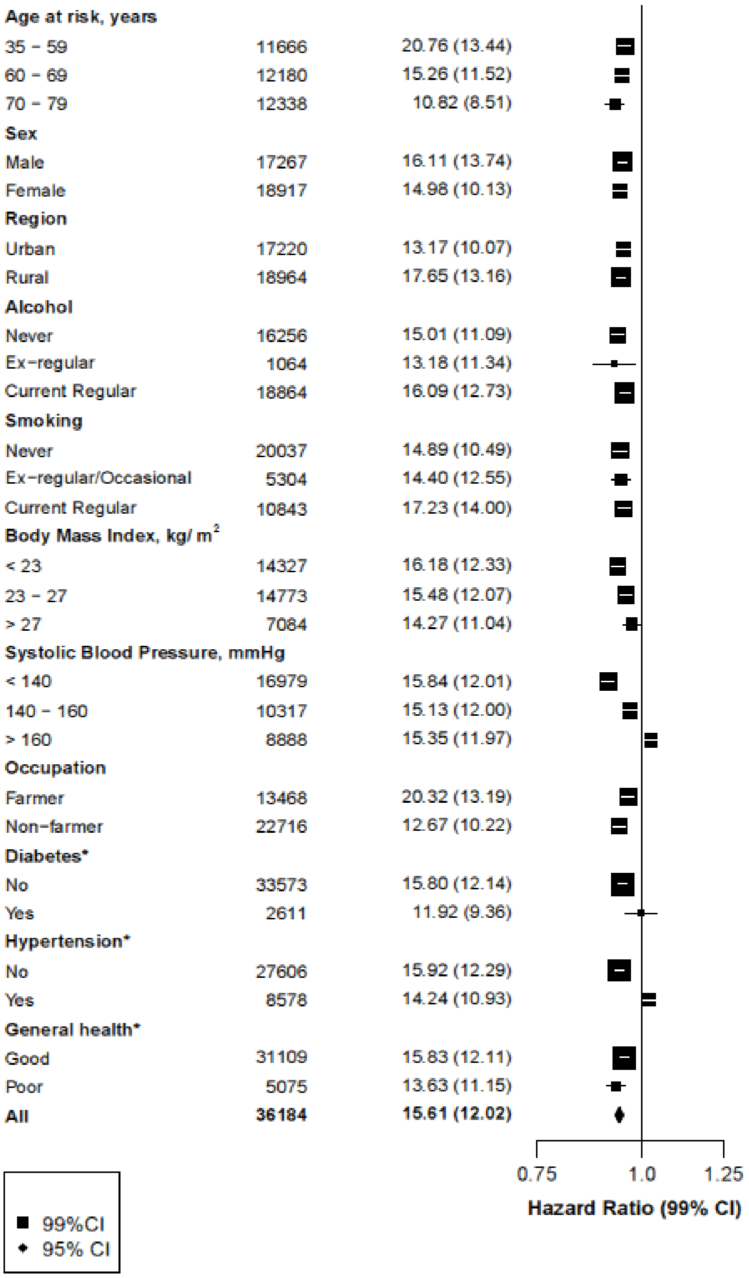

■ 99%CI
◆ 95% CI

© 2017 China Kadoorie Biobank Study Collaborative Group et al. *JAMA Cardiology*.

eFigure 13: Adjusted HRs for a) major coronary events and b) ischemic stroke per 4 MET−h/day higher usual total physical activity by baseline characteristics

Subgroup	a) Major coronary events: No. of cases	Mean (SD) MET−h/day	Hazard Ratio per 4 usual MET−h/day higher (99% CI)	χ^2_1 (P for trend or heterogeneity)	b) Ischemic stroke: No. of cases	Mean (SD) MET−h/day	Hazard Ratio per 4 usual MET−h/day higher (99% CI)	χ^2_1 (P for trend or heterogeneity)
**Age at risk, years**								
35 − 59	1254	21.88 (14.13)	0.97 (0.92,1.01)	21.9 (2.84E−06)	8427	20.11 (13.09)	0.96 (0.94,0.97)	1.5 (2.31E−01)
60 − 69	1539	14.28 (11.02)	0.88 (0.84,0.93)		8863	14.95 (11.35)	0.97 (0.94,0.99)	
70 − 79	2289	9.91 (7.80)	0.84 (0.79,0.90)		8357	10.68 (8.25)	0.97 (0.94,1.00)	
**Sex**								
Male	2902	14.88 (13.10)	0.93 (0.90,0.96)	12.4 (4.19E−04)	11542	15.86 (13.67)	0.96 (0.95,0.98)	0.1 (7.43E−01)
Female	2180	13.27 (9.37)	0.85 (0.80,0.90)		14105	14.76 (9.82)	0.96 (0.94,0.98)	
**Region**								
Urban	2175	12.23 (10.21)	0.93 (0.88,0.98)	2.5 (1.13E−01)	13871	13.01 (9.69)	0.96 (0.94,0.98)	0.9 (3.59E−01)
Rural	2907	15.65 (12.46)	0.90 (0.86,0.93)		11776	17.90 (13.27)	0.96 (0.95,0.98)	
**Alcohol**								
Never	2384	13.42 (10.51)	0.87 (0.83,0.91)	9.7 (1.89E−03)	11218	14.75 (10.73)	0.96 (0.94,0.98)	0.5 (5.00E−01)
Ex−regular	222	12.16 (10.06)	0.92 (0.81,1.05)		639	13.56 (11.46)	0.97 (0.91,1.04)	
Current Regular	2476	15.11 (12.75)	0.93 (0.90,0.97)		13790	15.74 (12.47)	0.96 (0.95,0.98)	
**Smoking**								
Never	2324	13.39 (10.15)	0.88 (0.84,0.93)	5.0 (2.52E−02)	14974	14.66 (10.16)	0.96 (0.94,0.98)	0.5 (4.85E−01)
Ex−regular/Occasional	815	12.37 (11.48)	0.88 (0.83,0.95)		3631	14.24 (12.43)	0.96 (0.93,0.99)	
Current Regular	1943	15.90 (13.15)	0.93 (0.89,0.97)		7042	17.05 (14.04)	0.97 (0.95,0.98)	
**Body Mass Index, kg/m^2**								
< 23	2232	14.59 (11.73)	0.89 (0.85,0.93)	6.7 (9.80E−03)	9204	16.06 (12.19)	0.96 (0.94,0.97)	7.9 (1.32E−02)
23 − 27	1946	14.08 (11.78)	0.92 (0.88,0.96)		11007	15.18 (11.75)	0.97 (0.95,0.99)	
> 27	904	13.42 (11.27)	0.96 (0.90,1.03)		5436	14.04 (10.72)	0.99 (0.96,1.01)	
**Systolic Blood Pressure, mmHg**								
< 140	2316	14.98 (12.16)	0.89 (0.85,0.92)	12.2 (4.69E−04)	12750	15.61 (11.67)	0.93 (0.92,0.95)	89.2 (1.87E−18)
140 − 160	1474	13.44 (11.46)	0.91 (0.87,0.96)		7240	14.96 (11.83)	0.99 (0.97,1.01)	
> 160	1292	13.62 (10.93)	0.97 (0.92,1.03)		5657	14.83 (11.69)	1.02 (1.00,1.05)	
**Occupation**								
Farmer	1809	19.47 (12.88)	0.94 (0.91,0.99)	3.8 (5.14E−02)	8612	20.12 (13.27)	0.97 (0.96,0.99)	3.7 (6.50E−02)
Non−farmer	3273	11.27 (9.80)	0.90 (0.86,0.94)		17035	12.79 (10.00)	0.96 (0.94,0.97)	
**Diabetes***								
No	4571	14.58 (11.93)	0.91 (0.89,0.94)	0.1 (7.17E−01)	23716	15.53 (11.88)	0.96 (0.95,0.97)	7.4 (7.55E−03)
Yes	511	10.66 (8.27)	0.93 (0.83,1.04)		1931	11.88 (9.04)	1.02 (0.97,1.07)	
**Hypertension***								
No	3844	14.74 (12.20)	0.91 (0.88,0.94)	3.3 (7.00E−02)	19643	15.64 (12.01)	0.96 (0.94,0.97)	53.3 (6.63E−12)
Yes	1238	12.46 (9.67)	0.95 (0.90,1.01)		6004	14.00 (10.66)	1.03 (1.01,1.06)	
**General health***								
Good	4220	14.58 (11.80)	0.92 (0.89,0.95)	2.2 (1.38E−01)	22432	15.46 (11.82)	0.96 (0.95,0.98)	0.7 (4.18E−01)
Poor	862	12.26 (10.82)	0.88 (0.83,0.95)		3215	13.85 (10.92)	0.97 (0.94,1.01)	
**All**	**5082**	**14.01 (11.55)**	**0.91 (0.89,0.93)**		**25647**	**15.39 (11.80)**	**0.96 (0.95,0.97)**	

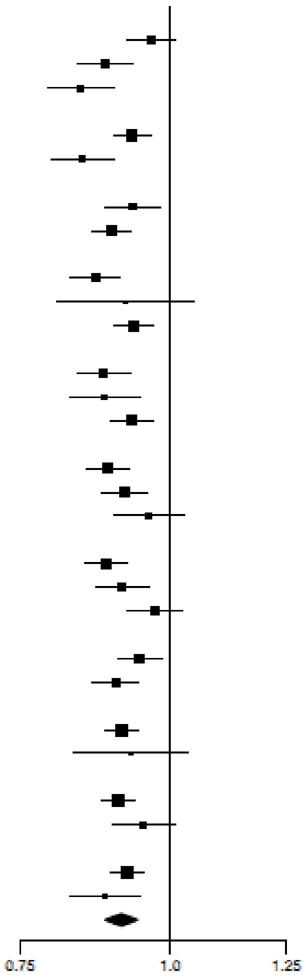

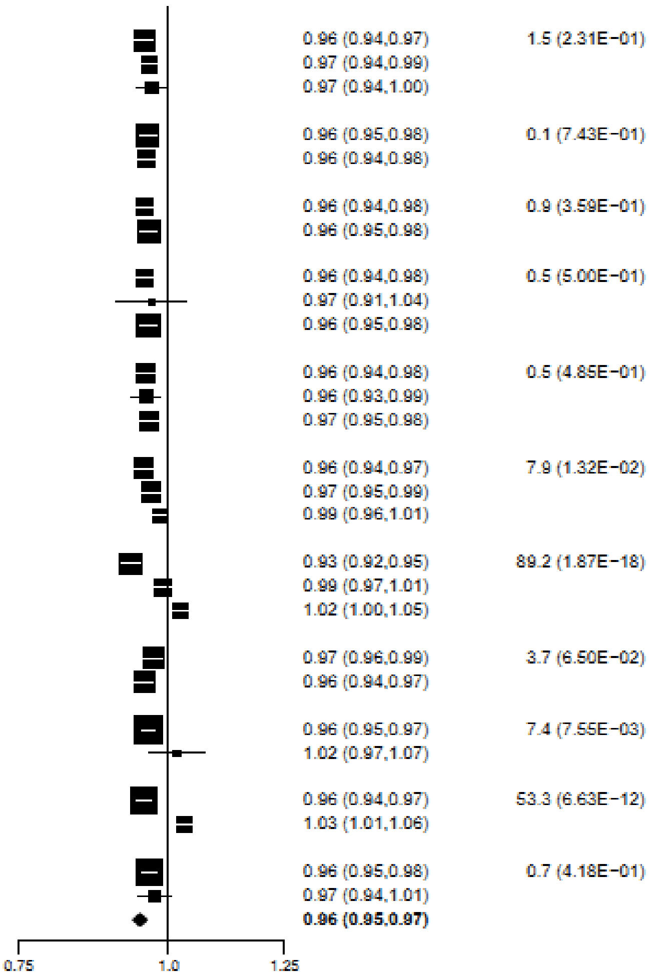

■ 99%CI
◆ 95% CI

© 2017 China Kadoorie Biobank Study Collaborative Group et al. *JAMA Cardiology.*

eFigure 14: Adjusted HRs for a) intracerebral hemmorhage and b) cardiovascular death per 4 MET−h/day higher total physical activity by baseline characteristics

a) Intracerebral hemmorhage

Subgroup	No. of cases	Mean (SD) MET−h/day	Hazard Ratio per 4 usual MET-h/day higher (99% CI)	χ^2_1 (P for trend or heterogeneity)
**Age at risk, years**				
35 − 59	1848	22.89 (14.09)	0.96 (0.93,1.00)	8.0 (4.75E−03)
60 − 69	1711	17.37 (12.36)	0.92 (0.88,0.97)	
70 − 79	1693	12.12 (9.88)	0.89 (0.84,0.95)	
**Sex**				
Male	2767	17.92 (14.19)	0.94 (0.91,0.97)	0.0 (9.74E−01)
Female	2485	17.29 (11.66)	0.94 (0.90,0.98)	
**Region**				
Urban	1185	15.53 (12.29)	0.99 (0.93,1.05)	7.6 (5.95E−03)
Rural	4067	18.23 (13.22)	0.92 (0.90,0.95)	
**Alcohol**				
Never	2551	17.24 (12.34)	0.92 (0.89,0.96)	2.3 (1.26E−01)
Ex−regular	200	13.18 (12.44)	0.86 (0.75,0.98)	
Current Regular	2501	18.37 (13.72)	0.95 (0.92,0.98)	
**Smoking**				
Never	2641	17.25 (12.04)	0.94 (0.90,0.97)	0.0 (9.58E−01)
Ex−regular/Occasional	803	16.48 (13.44)	0.93 (0.88,0.99)	
Current Regular	1808	18.67 (14.20)	0.94 (0.90,0.97)	
**Body Mass Index, kg/m^2**				
< 23	2642	17.80 (12.91)	0.92 (0.89,0.95)	1.7 (1.98E−01)
23 − 27	1831	18.08 (13.52)	0.96 (0.93,1.00)	
> 27	779	15.93 (12.32)	0.93 (0.88,1.00)	
**Systolic Blood Pressure, mmHg**				
< 140	1803	18.02 (13.32)	0.89 (0.85,0.92)	45.2 (1.77E−11)
140 − 160	1558	17.07 (12.89)	0.94 (0.90,0.98)	
> 160	1891	17.69 (12.94)	1.03 (0.99,1.07)	
**Occupation**				
Farmer	2845	21.45 (13.22)	0.95 (0.92,0.99)	2.9 (8.72E−02)
Non−farmer	2407	13.10 (11.31)	0.92 (0.88,0.96)	
**Diabetes***				
No	5032	17.78 (13.08)	0.93 (0.91,0.96)	4.7 (2.93E−02)
Yes	220	14.11 (12.17)	1.04 (0.92,1.18)	
**Hypertension***				
No	3891	18.12 (13.30)	0.93 (0.90,0.95)	20.1 (7.32E−06)
Yes	1361	16.21 (12.23)	1.02 (0.97,1.07)	
**General health***				
Good	4400	18.23 (13.16)	0.95 (0.93,0.98)	10.2 (1.41E−03)
Poor	852	14.49 (12.06)	0.87 (0.82,0.93)	
**All**	**5252**	**17.71 (13.00)**	**0.95 (0.93,0.97)**	

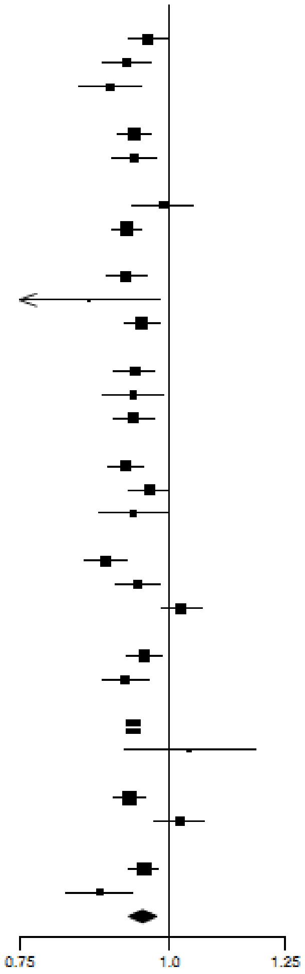

b) Cardiovascular deaths

Subgroup	No. of cases	Mean (SD) MET−h/day	Hazard Ratio per 4 usual MET-h/day higher (99% CI)	χ^2_1 (P for trend or heterogeneity)
**Age at risk, years**				
35 − 59	1754	22.00 (14.31)	0.91 (0.88,0.95)	17.9 (2.28E−05)
60 − 69	2487	15.72 (11.84)	0.87 (0.83,0.90)	
70 − 79	4196	10.73 (8.94)	0.83 (0.80,0.87)	
**Sex**				
Male	4643	14.81 (13.16)	0.88 (0.86,0.91)	8.0 (0.00)
Female	3794	14.22 (10.27)	0.84 (0.80,0.87)	
**Region**				
Urban	2419	11.78 (10.34)	0.86 (0.82,0.91)	0.2 (0.62)
Rural	6018	15.66 (12.37)	0.87 (0.85,0.90)	
**Alcohol**				
Never	4162	14.22 (11.28)	0.84 (0.81,0.87)	14.0 (1.84E−04)
Ex−regular	388	11.66 (9.79)	0.82 (0.74,0.92)	
Current Regular	3887	15.18 (12.77)	0.90 (0.87,0.92)	
**Smoking**				
Never	3944	14.07 (10.71)	0.85 (0.81,0.88)	7.1 (0.01)
Ex−regular/Occasional	1438	12.98 (11.74)	0.87 (0.82,0.91)	
Current Regular	3055	15.90 (13.35)	0.89 (0.86,0.92)	
**Body Mass Index, kg/m^2**				
< 23	4337	15.14 (12.03)	0.87 (0.84,0.89)	2.5 (1.16E−01)
23 − 27	2810	14.14 (12.04)	0.86 (0.83,0.90)	
> 27	1290	13.44 (11.37)	0.91 (0.86,0.96)	
**Systolic Blood Pressure, mmHg**				
< 140	3079	14.80 (12.31)	0.81 (0.78,0.84)	81.8 (1.53E−19)
140 − 160	2507	14.05 (11.62)	0.88 (0.85,0.92)	
> 160	2851	14.71 (11.84)	0.97 (0.93,1.00)	
**Occupation**				
Farmer	3895	19.32 (12.64)	0.91 (0.88,0.94)	15.5 (0.00)
Non−farmer	4542	10.45 (9.59)	0.85 (0.81,0.88)	
**Diabetes***				
No	7772	14.82 (12.09)	0.87 (0.85,0.89)	4.5 (0.03)
Yes	665	11.35 (9.68)	0.94 (0.86,1.03)	
**Hypertension***				
No	6256	14.97 (12.27)	0.86 (0.84,0.89)	21.7 (0.00)
Yes	2181	13.32 (10.91)	0.94 (0.90,0.98)	
**General health***				
Good	6663	15.18 (12.14)	0.90 (0.87,0.92)	18.4 (0.00)
Poor	1774	12.17 (10.91)	0.82 (0.78,0.86)	
**All**	**8437**	**14.38 (11.82)**	**0.87 (0.85,0.89)**	

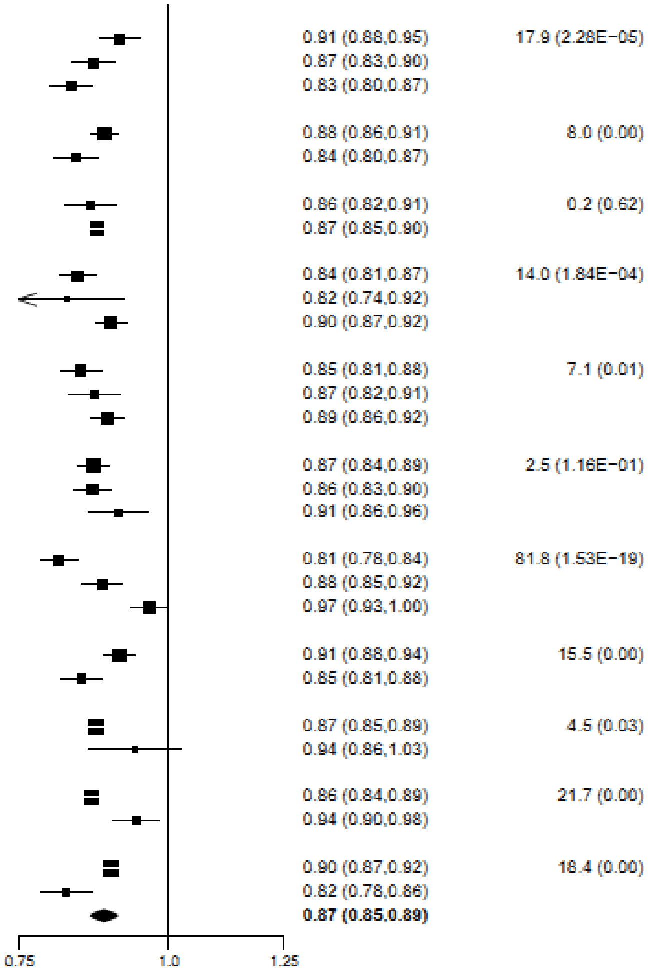

■ 99%CI
◆ 95% CI

© 2017 China Kadoorie Biobank Study Collaborative Group et al. *JAMA Cardiology.*

eFigure 15: Effect on HRs for major vascular events per 4 MET−h/day higher total physical activity sequential adjustment for potential confounders

Major vascular events (n = 36,184)

Adjustments	Hazard Ratio per usual 4 MET-h/day higher (95% CI)	χ^2_1
Age, sex and region	0.94 (0.93,0.95)	215.5
+ smoking and alcohol	0.95 (0.94,0.95)	189.6
+ income and education	0.94 (0.94,0.95)	193.3
+ fresh fruit	0.94 (0.94,0.95)	191.9
+ self-rated health	0.95 (0.95,0.96)	141.3
+ dietary factors[1]	0.95 (0.95,0.96)	138.5
+ dietary factors[2]	0.95 (0.95,0.96)	134.9
+ dietary factors[3]	0.95 (0.95,0.96)	137.0
+ respiratory measures[4]	0.95 (0.95,0.96)	137.0
+ sedentary leisure time	0.96 (0.95,0.96)	117.2
+ heart rate	0.96 (0.95,0.97)	105.0
+ body mass index	0.96 (0.95,0.97)	84.8
+ systolic blood pressure	0.97 (0.96,0.97)	73.4

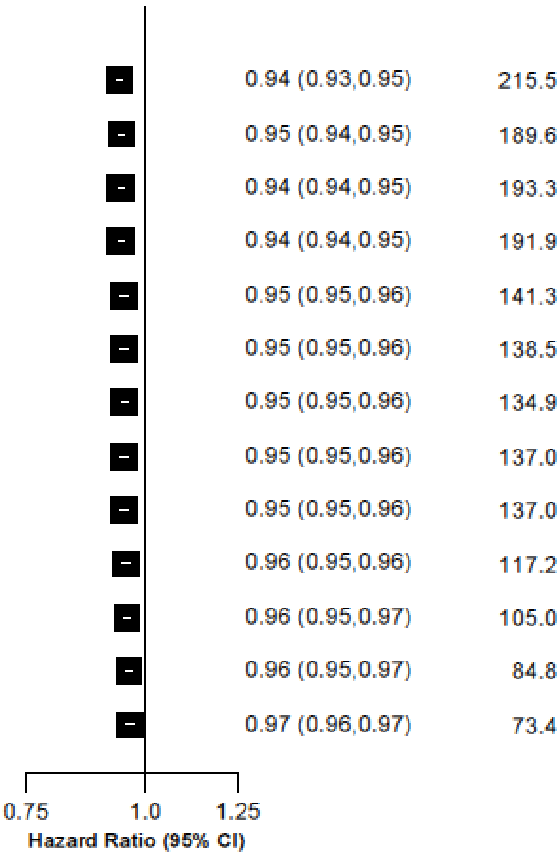

1: Rice, wheat, and staple foods,
2: Meat, poultry, fresh fish, eggs
3: Diary, soyabean, and preserved vegatables
4: Ratio of forced expiratory volume in 1 second to forced vital capacity

© 2017 China Kadoorie Biobank Study Collaborative Group et al. *JAMA Cardiology.*
